# Abortion decision-making process trajectories and determinants in low- and middle-income countries: A mixed-methods systematic review and meta-analysis

**DOI:** 10.1016/j.eclinm.2022.101694

**Published:** 2022-10-17

**Authors:** Paul Lokubal, Ines Corcuera, Jessica Macias Balil, Sandrena Ruth Frischer, Christine Nalwadda Kayemba, Jennifer J. Kurinczuk, Charles Opondo, Manisha Nair

**Affiliations:** a National Perinatal Epidemiology Unit, Nuffield Department of Population Health, University of Oxford, Oxford, UK; b Chelsea and Westminster Hospital, NHS Foundation Trust, London, UK; c MSI Reproductive Choices, London, UK; d Ethox Centre, Nuffield Department of Population Health, University of Oxford, Oxford, UK; e Department of Community Health and Behavioural Sciences, School of Public Health, College of Health Sciences, Makerere University, Kampala, Uganda; f Department of Medical Statistics, Faculty of Epidemiology and Population Health, London School of Hygiene and Tropical Medicine, London, UK

**Keywords:** Abortion, Pregnancy termination, Decision-making, Abortion trajectories, Low- and middle-income countries, Mixed-methods, Systematic review, Meta-analysis

## Abstract

**Background:**

About 45.1% of all induced abortions are unsafe and 97% of these occur in low- and middle-income countries (LMICs). Women's abortion decisions may be complex and are influenced by various factors. We aimed to delineate women's abortion decision-making trajectories and their determinants in LMICs.

**Methods:**

We searched Medline, EMBASE, PsychInfo, Global Health, Web of Science, Scopus, IBSS, CINAHL, WHO Global Index Medicus, the Cochrane Library, WHO website, ProQuest, and Google Scholar for primary studies and reports published between January 1, 2000, and February 16, 2021 (updated on June 06, 2022), on induced abortion decision-making trajectories and/or their determinants in LMICs. We excluded studies on spontaneous abortion. Two independent reviewers extracted and assessed quality of each paper. We used “best fit” framework synthesis to synthesise abortion decision-making trajectories and thematic synthesis to synthesise their determinants. We analysed quantitative findings using random effects model. The study protocol is registered with PROSPERO number CRD42021224719.

**Findings:**

Of the 6960 articles identified, we included 79 in the systematic review and 14 in the meta-analysis. We identified nine abortion decision-making trajectories: pregnancy awareness, self-reflection, initial abortion decision, disclosure and seeking support, negotiations, final decision, access and information, abortion procedure, and post-abortion experience and care. Determinants of trajectories included three major themes of autonomy in decision-making, access and choice. A meta-analysis of data from 7737 women showed that the proportion of the overall women's involvement in abortion decision-making was 0.86 (95% CI:0.73–0.95, I^2^ = 99.5%) and overall partner involvement was 0.48 (95% CI:0.29–0.68, I^2^ = 99.6%).

**Interpretation:**

Policies and strategies should address women's perceptions of safe abortion socially, legally, and economically, and where appropriate, involvement of male partners in abortion decision-making processes to facilitate safe abortion. Clinical heterogeneity, in which various studies defined “the final decision-maker” differentially, was a limitation of our study.

**Funding:**

Nuffield Department of Population Health DPhil Scholarship for PL, University of Oxford, and the Medical Research Council Career Development Award for MN (Grant Ref: MR/P022030/1).

**Research in context**

**Evidence before this study**

In low- and middle-income countries (LMICs), women's abortion decisions may follow complex and cyclical multiphasic trajectories that are influenced by various structural, health system, interpersonal and individual factors, but there is no critical appraisal of such evidence. We conducted a systematic review of published and unpublished literature from January 1, 2000 to June 06, 2022 using the following key search terms: “abortion,” “decision-making”, and “developing countries”. A total of 6960 articles were identified of which 79 were included in the systematic review and 14 in the meta-analysis.

**Added value of this study**

The systematic review and meta-analysis identified nine complex and inter-linked components which constitute abortion decision-making trajectories in LMIC settings and their multifactorial determinants highlighting: (i) varying levels of women's autonomy in the decision-making process, (ii) the role and influence of male partners, and (iii) the role of women's perceptions of abortion safety in shaping their abortion decision-making trajectories. Although overall women's involvement in abortion decision-making was 86% (95% CI: 73–95%, I^2^=99.5%), they were primary decision makers in 53% (95% CI: 34–73%, I^2^=99.7%) while the overall male partner's involvement was 48% (95% CI: 29–68%, I^2^=99.6%).

**Implications of all available evidence**

Policies and strategies should address women's perceptions of safe abortion socially, legally, and economically, and where appropriate, involvement of male partners in abortion decision-making processes to facilitate safe abortion. Future research into women's perceptions of abortion safety and the role of male partners in the abortion decision-making trajectories in LMICs is required. Further research is also needed to understand how the broad trajectories framework developed through this review apply to different groups of women such as rape victims, commercial sex workers, refugees, adolescents, and women living with HIV for which medical abortion is not indicated.

Alt-text: Unlabelled box

## Introduction

Globally, each year between 2015 and 2019, an estimated 48% (121 million) of all pregnancies were unintended and 61% (73 million) of these ended in induced abortion.^1^^,^^2^ Globally, each year between 2010 and 2014, about 45.1% of all abortions were unsafe and 97% of these occurred in low- and middle-income countries(LMICs).^3^

The circumstances surrounding a woman's decision to seek an abortion can be time-specific and variable.^4^ Due to the socioeconomic and power dynamics involved in abortion,^5^ abortion decision-making trajectories are often complex, iterative, multiphasic, dynamic, context-specific, and may involve periods of intense negotiations between the woman and significant others.^4^^,^^6^, ^7^, ^8^, ^9^, ^10^, ^11^ Women may “suffer in silence” due to the uncertainty about the decision to terminate a pregnancy and other people's reaction to the decision.^12^ The abortion trajectories chosen may influence abortion outcome and access to post-abortion care.^6^^,^^12^ The particular trajectory taken is influenced by various legal, socioeconomic, demographic, and cultural factors such as financial stability, relationship stability, the influence of significant others, risk perceptions, stigma, knowledge of abortion laws, and availability and access to abortion services including misoprostol.^4^^,^^6^, ^7^, ^8^, ^9^, ^10^ While there is some understanding of women's decision-making processes for seeking abortion care, there is no critical appraisal of such evidence through a systematic review and meta-analysis to map out the complex abortion decision-making trajectories and their determinants in LMICs, which have a high burden of unsafe abortions and their complications. We used the definition of Coast et al. (2018) for abortion decision-making trajectories as “*the processes and transitions occurring overtime for a pregnancy that ends in abortion”*.^4^ In this review, we also included the post-abortion trajectories since the abortion decision-making process spans the pre-abortion phase through the abortion experience to the post-abortion period including management of post-abortion complications and post-abortion contraception.^13^

In this systematic review, we aimed to critically synthesise existing evidence on abortion decision-making trajectories and their determinants in LMIC contexts to inform policy and further research on strategies to reduce unsafe abortion rates and consequent unsafe abortion-related morbidity and mortality.

## Methods

### Search strategy and selection criteria

We followed the preferred reporting items for systematic reviews and meta-analyses protocol (PRISMA-P) 2015 statement^14^ in developing the protocol.^15^ The protocol is registered with the international prospective register of systematic reviews (PROSPERO), registration number CRD42021224719.

We searched the following electronic bibliographic databases and grey literature sources: Ovid Medline, Ovid EMBASE, Ovid PsychInfo, Ovid Global Health, Web of Science (including Social Science Citation Index), Scopus, IBSS, CINAHL via EBSCO, WHO Global Index Medicus, the Cochrane Library ProQuest, Google Scholar, and the WHO website. The Library Manager helped with identifying relevant search terms which comprised the following three key concepts and their synonyms: “abortion,” “decision-making”, and “developing countries” which were combined with Boolean operators. We modified the search terms depending upon the database and used both keywords and medical subject headings (MeSH) in the search process. We used the search filters for LMICs from the Cochrane filter (https://epoc.cochrane.org/lmic-filters). We also searched reference lists of all included studies for possible relevant studies that may have been missed in the earlier searches. The full search strategy for Ovid Medline is in the supplementary materials.

We included published and unpublished primary observational, intervention, and qualitative studies and reports that reported on either the women's or male partners’ decision-making trajectories or their determinants or both for induced abortions in LMICs. We excluded studies that focused on spontaneous abortion. We restricted the search to studies published between January 1, 2000, and February 16, 2021, with no language restrictions. We updated the searches on June 06, 2022. The period from 2000 was chosen because it marked the start of the millennium development goals (MDGs). We also wanted to capture the contemporary studies on abortion decision-making in LMICs.

The first author (PL) screened and selected all articles based on titles and abstracts while IC, JM, and SF were the second independent reviewers. We resolved discrepancies through discussion and/or by involving the other authors (CN, JK, CO, MN).

We extracted the following information: study authors, study aim(s); study setting (including location(s) and year(s) of publication); inclusion/exclusion criteria and participant characteristics; study methodology including study design, sample size, data collection, and analytical methods; results including numbers and proportions of various final decision-makers on abortion, themes, first order quotes (study participants’ quotes verbatim), second order quotes (author interpretations/explanations); strengths and limitations; and all information needed to assess the risk of bias. We generated separate data extraction templates for qualitative and quantitative findings. At least two reviewers independently extracted data from each included study.

We assessed risk of bias for quantitative studies using the Joanna Briggs Institute (JBI) assessment tool^16^^,^^17^ and used the critical appraisal skills programme (CASP) appraisal checklist for qualitative studies.^18^ We resolved disagreements in the quality assessment findings through consensus. We did not exclude any study based on quality assessment alone.^19^ At least two reviewers independently conducted quality assessment for each included study.

### Data analysis

We analysed qualitative and quantitative studies separately and integrated the findings using the convergent synthesis approach suggested by Hong et al.^20^ We adopted the “best fit” framework synthesis^21^ using “the trajectories of women's abortion-related care” conceptual framework developed by Coast et al.^4^ We compared and mapped extracted information onto Coast's framework, adding or modifying the trajectories based on the extracted data. To analyse the determinants of abortion decision-making trajectories, we used the thematic synthesis approach proposed by Thomas and Harden.^22^ The synthesis involves three overlapping stages: developing coding schemes; developing descriptive themes from the coding schemes; and generating analytical themes from the descriptive themes.^22^ We conducted sensitivity analyses to examine if themes synthesised from the qualitative studies varied by the graded quality of the included papers.^22^ For the quantitative synthesis, we categorised abortion decision-making into decisions made by the woman alone (solely or primarily – depending on how this had been ascertained in the primary study), partner (solely or primarily), jointly (woman and partner), and others (solely or primarily). We then created two additional categorical variables: “overall woman involvement” was generated by summing woman alone and joint decision-making and “overall partner involvement” was created by summing partner alone and joint decision-making. We conducted meta-analyses of proportions using the random-effects model that assumed between-study heterogeneity for the variables ‘woman alone’, ‘overall woman involvement’, and ‘overall partner involvement’. We tested for heterogeneity using the I^2^, Tau square, and the Q-statistic. To investigate potential sources of heterogeneity, we conducted subgroup analyses by study setting's abortion laws, income status, and geographical location. As recommended by Barker et al. (2021), we did not test for publication bias as the tests would not be appropriate for meta-analysis of proportions.^23^ Tests for publication bias are primarily for comparative data and assumes that studies with positive results are more likely to be published than those with negative results. Therefore, for single proportions, with no comparator, such as incidence/prevalence studies,^23^ and in our case the proportion of involvement of various actors in the abortion decision making process, the tests for publication bias were not appropriate. We also conducted sensitivity analyses by quality of included papers (low, medium, high), and study setting (community or hospital cases). Following recommendations from the Cochrane Qualitative and Implementation Methods Group,^24^ we assessed the confidence in the evidence of the recommendations that we generated using the Grades of Recommendation, Assessment, and Evaluation – Confidence in Evidence from Reviews of Qualitative Research (GRADE-CERQUal) approach.^25^ We assessed each CERQUal component separately, namely, methodological limitations, coherence, adequacy and relevance of the qualitative evidence synthesis to assign a level of confidence to each recommendation: no or very minor concerns for those that were unlikely to reduce confidence in the findings, minor concerns for those that may reduce confidence, moderate concerns for those that would probably reduce confidence and serious concerns for those that were likely to reduce confidence.^25^ Full details are included in the study protocol.^15^ We did not require ethical approval for this systematic review and meta-analysis.

### Role of the funding source

The funders had no role in study design; in the collection, analysis, and interpretation of data; in the writing of the report; and in the decision to submit the paper for publication. PL, IC, JM, and SF had full access to all the information for the paper and have verified all data included in the review. All the authors approved the final version to be submitted while PL had the final responsibility for the decision to submit for publication.

## Results

**Figure 1:**
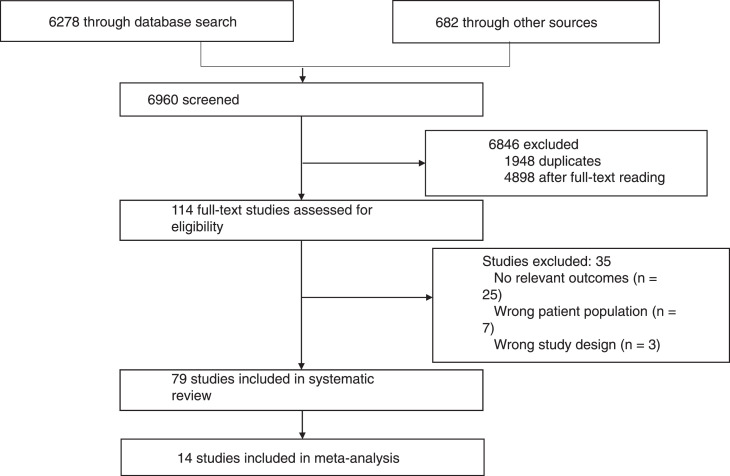
The PRISMA chart showing exclusion and inclusion of studies.

The final search yielded a total of 6960 studies. After deduplication, we screened title and abstract of 4269 studies and included 113 studies for full text screening. We excluded 34 studies following full text and extracted and analysed the remaining 79 studies as shown in Figure 1. The details of excluded articles are in the supplementary material.

**Figure 2:**
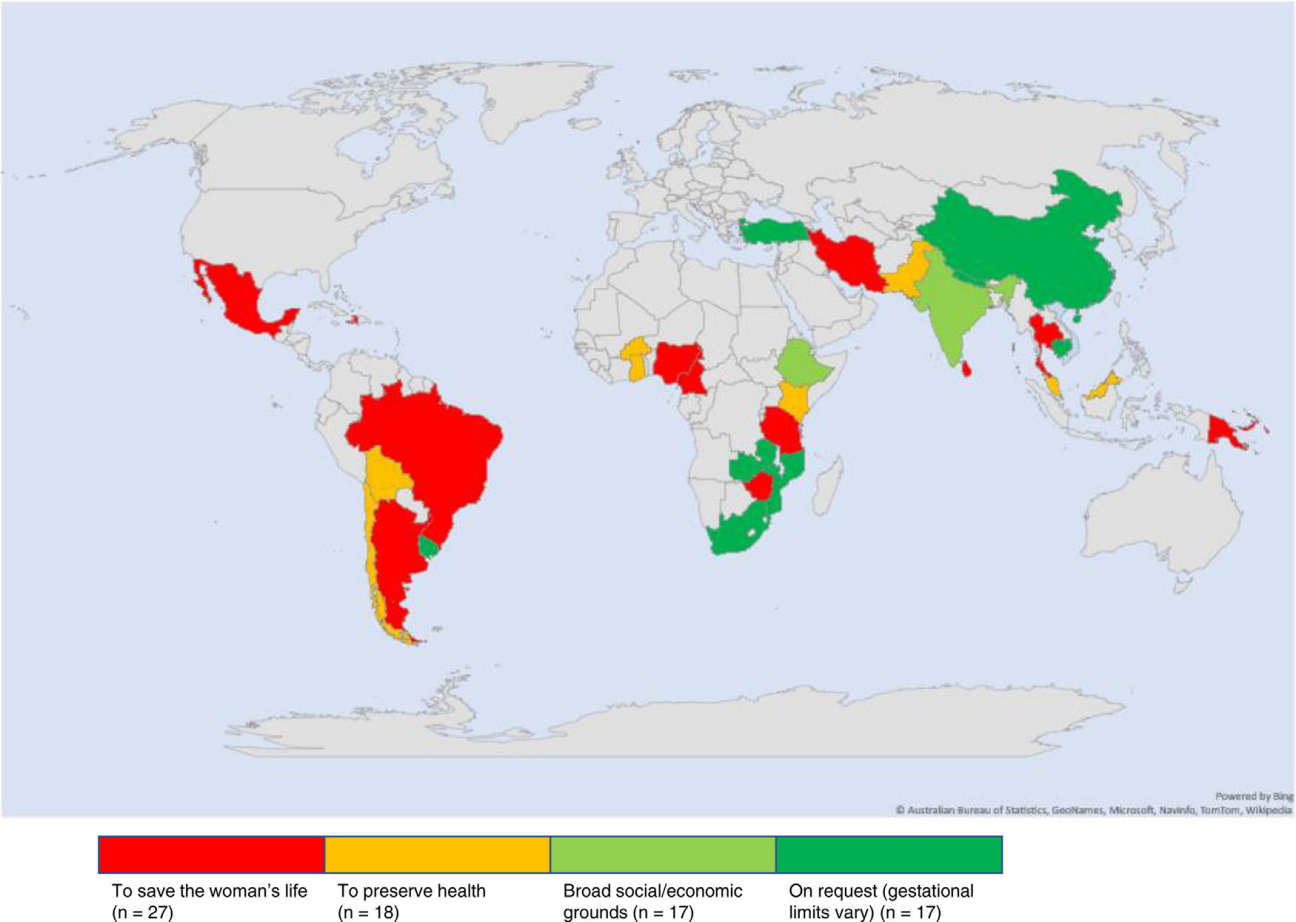
Map showing included studies' settings and abortion laws.

The Study settings and their abortion laws are shown in Figure 2. Thirty-three of the studies were from sub-Saharan Africa (SSA), 28 from Southern and Eastern Asia, 13 from Latin America, two from Eastern Europe, one each from the Pacific and Caribbean Islands while one study included countries from both SSA and Latin America. The included studies are summarised in Table 1. Forty-seven studies were qualitative in design, 14 used mixed-methods, 17 were quantitative cross-sectional, and one was a partially randomised controlled trial. Overall, 52 of the articles were high quality, eight medium quality, and 19 were found to be of low quality (see supplementary materials).

**Table 1:** Summary of included studies (*N*=79).

Study ID	Country of setting	Study Designs	Sample size	Participant characteristics	Study setting (place, rural/urban)	Data collection methods	Quality assessment
Coast et al (2016)^6^	Zambia	Mixed methods: in-depth interviews; cross-sectional study	112	Adolescents and women aged 15–43 presenting for care at emergency ward following unsafe abortion	City Hospital; Urban	Semi-structured and structured questionnaires	High
Freeman et al (2017)^7^	Zambia	Qualitative: in-depth interviews	112	Women who received a safe abortion or care following an incomplete (unsafe) abortion	Hospital; Urban	Open-ended and supplementary closed-ended questionnaires	High
Ouedraogo et al (2020)^8^	Burkina Faso	Qualitative: ethnography	31 women and 5 men	Women seeking abortion; men whose partners had got abortion	clinic	In-depth interview questionnaires	High
Puri et al (2007)^9^	Nepal	Mixed methods: cross-sectional surveys; in-depth interviews	1496 - for quantitative; 30 for qualitative	Household survey of married women, 15-24 years; and married men, 15-27 years	households	Structured questionnaires	High
Bain et al (2019)^10^	Ghana	Qualitative: in-depth interviews	53	Female adolescents: had either continued a pregnancy to term, or had past experience of at least one self-induced abortion; additional interviews were conducted with purposively selected stakeholders	Clinic; Urban	Semi-structured	High
Loi et al (2018)^12^	Kenya	Qualitative: in-depth interviews	9	Women over 18 years; had induced abortion; received post-abortion care; gave consent	Hospital; Public	Semi-structured interview questionnaires	High
Oyeniran et al (2019)^26^	Nigeria	Qualitative: in-depth interviews	31 women	Had induced abortion; admitted to hospital	Hospital; Urban	Semi-structured guides	High
Vallely et al (2015)^27^	Papua New Guinea	Qualitative: in-depth and key informant interviews	28 IDIs, 8 KIIs	IDIs - women managed for complications of induced/incomplete abortion; Key informants - extensive experience working in both the government and church health services and non-governmental organisations (NGOs)	Hospital, Urban	Interview guides	High
Solheim et al (2020)^28^	Tanzania	Qualitative: ethnography, focus groups, in-depth interviews	198	Women with medical abortion after unwanted pregnancy; health workers; drug providers; college and university students; representatives from NGOs, district authorities and ministry; and drugstores	Clinic; drugstores; health facilities; urban	Interview guides	High
Baum et al (2020)^29^	Poland, Brazil, Nigeria	Qualitative: in-depth interviews	30	Aged 18 or older; speak English, Polish, or Portuguese	Online	Interview guide	High
Juarez et al (2011)^30^	Mexico	Qualitative: in-depth interviews	26	Aged 18-35; had at least one pregnancy; consented	Clinic: Urban	Semi-structured guide	High
Peres et al (2006)^31^	Brazil	Qualitative: retrospective narrative biographies; key informant interviews	123	Youth; key informants	Urban	Semi-structured interviews	Low
Srivastava et al (2019)^32^	India	Qualitative: in-depth interviews	40	Medical abortion users and their partners	Clinic	Semi-structured questionnaires	High
Tatum et al (2012)^33^	Mexico	Qualitative: in-depth interviews and focus group discussions	24	Adolescents; 13-17 years at time of pregnancy; became pregnant after the April 2007 law change; resident of Mexico City during the first 12 weeks of pregnancy; belonged to middle, lower-middle, or lower socioeconomic class; terminate or did not terminate pregnancy	Private secure rooms; urban	Semi-structured question guides	High
Ituarte et al (2021)^34^	Uruguay	Qualitative: in-depth interviews	14	Adolescents between 17 and 19 years of age who voluntarily terminated a pregnancy in public health services	Urban, health facilities	Interview guide	Moderate
Larrea et al (2021)^35^	Chile	Qualitative: in-depth interviews	11	Women who had accessed abortion services	Urban	Interview guide	High
Ferrari et al (2020)^36^	Brazil	Qualitative: in-depth interviews	10	Adolescents between 15 and 17 years, live in a favela (slum), had illegal abortion between ages 12 and 17.	Slum, urban	Interview guide	High
Ganatra et al (2002)^37^	India	Mixed methods: focus group discussions; Key informant interviews; cross-sectional surveys	1717 married women; 197 adolescents; 159 abortion providers	Women who had induced abortion during the study period (18 months) from 1996 to 1998 whether married or not; abortion providers in the study area and nearby towns	Clinic	Structured and semi-structured questionnaires	Medium
Penfold et al (2018)^38^	Kenya	Qualitative: in-depth interviews	22	Women who had received an abortion or post-abortion care service at selected clinics	Private Clinics: rural and urban	Structured and semi-structured interview questionnaires	Medium
Bui et al (2011)^39^	Vietnam	Qualitative: ethnography	20	HIV positive woman with unwanted pregnancy, either carried to term (7) or terminated (13)	Urban	Ethnographic notes	Moderate
Sri B. et al (2015)^40^	India	Qualitative: in-depth interviews	15 women	Women receiving medical abortion at the clinic	Rural/urban; clinic	Semi-structured questionnaires	High
Frederico et al (2018)^41^	Mozambique	Qualitative: in-depth interviews	14	Women aged 15-24; had had abortion	Community; Urban Centres	Semi-structured interview guideline	Low
Kumi-Kyereme et al (2014)^42^	Ghana	Mixed methods: in-depth interviews; Cross-sectional surveys	401 for cross-sectional; 35 for IDI	Accredited abortion providers; women who had undergone an abortion between January and December 2010.	Hospital; Urban	Structured and semi-structured questions	High
Ramachandar et al (2004)^43^	India	Qualitative: key informant; in-depth interviews	97 women who had abortions and 18 village health nurses as KIIs	Married women who had abortions in the previous six months; village health nurses as key informants	Clinics; Remote to peri-urban	Semi-structured questionnaires	Low
Chiweshe et al (2021)^44^	Ethiopia, Malawi, Zambia	Qualitative: in-depth interviews	133	Adolescents aged 10–19 years, either seeking a safe abortion or had come for post-abortion care	Clinics	Interview guides	High
Heilborn et al (2012)^45^	Brazil	Qualitative: ethnography and in-depth interviews	28 (13 men, 15 women)	Young people, aged 18-27, living in Rio de Janeiro, and had experiences of contraception, unforeseen pregnancies and abortion	Urban	Interview guides	Low
Osur et al (2015)^46^	Kenya	Mixed methods: focus group discussions; key informant interviews; cross-sectional surveys	320 for cross-sectional; 21 KIIs; 2 FGDs	Women with unsafe abortion treated for complications; community health workers; pharmacists; community representatives (teachers and women leaders)	Clinic	Structured questionnaires and interview guides	High
Dahlbäck et al (2010)^47^	Zambia	Mixed methods: cross-sectional and in-depth interviews	87 women	Admitted with a diagnosis of incomplete abortion; had undergone an MVA; hemodynamically stable; given informed consent	University Teaching Hospital, Lusaka, city	Semi-structured and structured questionnaires	Low
Masuda et al (2020)^48^	Cambodia	Qualitative: ground-up exploratory study	29: 16 women and 13 providers	Women: factory workers, aged 18 and above, seeking abortion services (medical abortion pills or surgical abortion); Providers: three providers were working at facilities where women were recruited, other providers were purposefully selected to involve a variety of types of facilities and providers.	clinics	Semi-structured questionnaires	High
Lima et al (2020)^49^	Brazil	Qualitative: in-depth interviews	8 adolescent girls	Adolescent girls going to school; seeking abortion	Urban, schools	Semi-structured questionnaires	Low
Marlow et al (2014)^50^	Kenya	Qualitative: focus group discussions	10 FGDs	Married and unmarried women; whether in school or not	Urban; rural	Topic guides	Low
Tong et al (2012)^51^	Malaysia	Qualitative: in-depth interviews	31 women	Women attending an urban family planning clinic, aged 21 and above and having had an induced abortion	Clinic; Urban	Structured and Semi-structured questionnaires	Low
Nourizadeh et al (2020)^52^	Iran	Qualitative: in-depth interviews	29	Women aged 15-48 with an unwanted pregnancy; husbands of women who had unwanted pregnancy; midwives; gynaecologists	Clinic patients	Semi-structured questions	High
Arambepola et al (2014)^53^	Sri Lanka	Mixed methods: in-depth interviews; unmatched case-control	671 women (171 cases; 600 controls)	Cases were women in the selected hospitals with complications following an unsafe abortion; Controls were mothers in postnatal wards following the delivery of an unintended pregnancy carried to term.	Hospital Patients	Structured and semi-structured questionnaires	High
Arnott et al (2017)^54^	Thailand	Qualitative: in-depth interviews	14	Women seeking abortion	Clinic; Urban	Semi-structured interview guides	High
Berry-Bibee et al (2018)^55^	Haiti	Mixed methods: in-depth interviews; focus group discussions; cross-sectional survey	8 FGDs (*n*=62); 13 IDIs; 255 = cross-sectional	Women seeking abortion; at least 18 years old; current or recent (6 weeks or less post-pregnancy) pregnancy at 20 or less weeks gestation (via self-report); Haitian Creole speakers; women's health care informants - community health workers, herbalists, traditional birth attendants, nurses, and physicians	University Hospital; Urban	Semi-structured and structured questionnaires	High
Bury et al (2012)^56^	Bolivia	Mixed methods: in-depth interviews; focus group discussions; cross-sectional survey	1386 for the survey; 115 for FGDs, 50 IDIs	For the survey: women aged 15-49 from Demographic and health Survey; For IDIs: women who accessed PAC in five public hospitals.	low-income peri-urban areas of 5 Bolivian cities	focus group discussions, in-depth interviews and structured survey questionnaires, semi-structured questionnaires	Low
Ganatra et al (2010)^57^	India	Qualitative: in-depth interviews	63	Women attending two clinics for medical abortion; consented	Clinics; urban and rural	In-depth open-ended questions	High
Gresh et al (2014)^58^	South Africa	Qualitative: In-depth interviews	20	Female university students aged less than 30 years	University; Urban	In-depth open-ended questions	Low
Jejeebhoy et al (2010)^59^	India	Mixed methods: in-depth interviews; cross-sectional survey	795 (26 IDIs)	Consenting; aged 24 or younger; not had a previous live birth, irrespective of marital status	Clinic	Interview guide	Low
Schuster (2005)^60^	Cameroon	Qualitative: in-depth interviews	65	Women treated for complications at hospitals; or history of abortion	Hospital; Urban	Semi-structured questionnaires	Low
Chareka et al (2021)^61^	Zimbabwe	Qualitative: in-depth interviews	198; 30 FGDs, 41 IDIs	Being female, self-identifying as selling sex and being between the ages 16–24 years	Urban and peri-urban	Interview guides; Semi-structured questionnaires	High
Harries et al (2021)^62^	South Africa	Qualitative: in-depth interviews	15	Women who had accessed abortion outside the formal system	Urban, various private places	Interview guide	High
Katz et al (2022)^63^	Nigeria	Qualitative: in-depth interviews	25	Clients at least 15 years old, had had abortion within the preceding 3 months	Clinic, urban	Semi-structured questionnaires	High
Calves et al (2002)^64^	Cameroon	Cross-sectional survey	384	Young women and men	Urban	Survey registers	High
Thapa et al (2013)^65^	Nepal	Cross-sectional survey	1172	Women receiving abortion services at the clinic;	Clinics; Urban and rural	Structured questionnaires	Low
Chunuan et al (2012)^66^	Sri Lanka	Cross-sectional survey	402	Women of any age who had an abortion, regardless of chronological age, gestational age, or type; and admitted to one of the study site hospitals.	Hospital	Structured self-report questionnaire	High
Banerjee et al (2014)^67^	India	Pre-post survey	2543	Married women between the ages of 15 and 45 and married men between the ages of 18 and 49	Health facilities	Semi-structured questionnaires, health registers	High
Zuo et al. (2015)^68^	China	Cross-sectional survey	1271	Unmarried women, aged 15–24 years.	Clinic	Structured questionnaires	High
Bui et al. (2010)^69^	Vietnam	Cross-sectional survey	707	HIV positive women accessing services through community health centres in the study areas	Clinic; rural	structured interview questionnaires	Low
Korejo et al. (2003)^70^	Pakistan	Cross-sectional survey	57	Women with history of attempted induced abortion admitted to hospital	Hospital, urban	Structured questionnaires; hospital records	Low
Zavier et al. (2020)^71^	India	Secondary data from community-based survey/cross-sectional survey	166	Women who had induced abortion in the two years preceding the survey	Urban and rural	Survey questionnaires	Medium
Dhillon et al. (2004)^72^	India	Cross-sectional survey	1851	Married women, aged 15–45	households	Survey/structured questionnaires	Medium
Munjial et al. (2006)^73^	India	Cross-sectional survey	31	Women who obtained abortion in the 5 years preceding the survey in the study area	Community	Structured questionnaire	Low
Azmat et al. (2012)^74^	Pakistan	Qualitative: exploratory, in-depth interviews, focus group discussions, questionnaires	8 FGDs, 15 IDIs, 76 exit interviews	Women with complications related to miscarriage and unsafe or incomplete abortions, and cases referred by reproductive health volunteers	Clinic	Semi-structured, topic guides, exit structured questionnaires	Moderate
Kebede et al. (2018)^75^	Ethiopia	Qualitative: ethnography, individual interviews, focus group discussions, analysis of cultural and social context	25 young women - 68 IDIs; 34 KIs; 144 persons for 12 FGDs	Age 18 -24 years; residing in Addis Ababa; never been married; had undergone medically unsafe abortions that had led to serious complications	Clinic, community; Urban	Semi-structured questionnaires	High
Dijk et al. (2011)^76^	Mexico	Qualitative: in-depth interviews	25	Women aged 18+; had safe abortion	Clinic; Urban	Interview guide	High
Dahlbäck et al. (2007)^77^	Zambia	Mixed methods: in-depth interviews and cross-sectional surveys	34 adolescent girls	Girls who had undergone unsafe abortion; received manual vacuum aspiration (MVA) at hospital; hemodynamically stable; given informed consent	University teaching hospital; urban/city	Semi-structured and structured questionnaires	High
Geressu et al. (2010)^78^	Ethiopia	Mixed methods: in-depth interviews and cross-sectional surveys	27 - women who had abortion; 512 health care providers	Women who had second trimester abortion in the public hospitals	29 hospitals countrywide	Semi-structured questionnaires	High
Izugbara et al. (2015)^79^	Kenya	Qualitative: in-depth interviews	50	Women who had induced or attempted to induce abortion; gave consent	Clinic; Urban and rural	Interview guide	High
Koster (2010)^80^	Nigeria	Qualitative: ethnography	652	Yoruba women in Lagos State	Clinics and community; Rural and urban	Participant observation, in-depth interview questionnaires, focus groups topic guides	High
Mitchell et al. (2010)^81^	Mozambique	Mixed methods: in-depth interviews; focus group discussions; cross-sectional survey	1661	Pregnant women 18–49 years of age (and adolescents 13–17 with parental consent); confirmed gestations of 6–11 weeks of pregnancy; requesting voluntary termination of pregnancy.	Hospital; Urban	Semi-structured questionnaires; exit interview questionnaires	High
Ramos et al. (2015)^82^	Argentina	Qualitative: in-depth interviews	24	Women who had had abortion with misoprostol at home; sought counselling or presented with symptoms of incomplete abortion at a public hospital in the city of Buenos Aires.	Hospital; urban	In-depth interview schedule	High
Chahal et al. (2017)^83^	Pakistan	Qualitative: ethnography	37 women	Abortion seeking women; abortion providers	Urban; Clinic	Semi-structured interviews; topic guides; participant observation	High
Rominski et al. (2017)^84^	Ghana	Qualitative: in-depth interviews and focus group discussions	29 - in-depth interviews; 8 focus groups of community members	Women receiving treatment for complications from a self-induced abortion or coming for an elective induced abortion; community members; consented	Hospital for IDIs; community/markets for FGDs	Topic guides for focus group discussions	High
MacFarlane et al. (2017)^85^	Turkey	Qualitative: in-depth interviews	14	Aged 18 years or older; obtained abortion services in Istanbul on/after January 1, 2009; fluent in Turkish or English	City; Urban	Interview guide	High
Gbagbo (2020)^86^	Ghana	Mixed methods: in-depth interviews and cross-sectional	401 questionnaires; 21 IDIs	All women who had induced abortion in Accra from January to December 2018; willing to be part of the study.	City; clinics	Structured and semi-structured questionnaires	High
John Lekan et al. (2017)^87^	Nigeria	Qualitative: in-depth interviews	40 male and female participants	Female and male; had abortion or procured services by partner; University students	University; Urban	Interview guides	Low
Rogers et al. (2019)^88^	Nepal	Qualitative: exploratory - Assets Focused Rapid Participatory Appraisal (AFRPA)	20	Women aged 15–49 years; obtained medical abortion pills for the termination of a pregnancy.	Clinic/pharmacy	Semi-structured questionnaires	High
Herrera et al. (2002)^89^	Mexico	Qualitative: Unclear	Unclear; but 12 women who had sought abortion; 1 priest, 2 doctors, gynaecologists and nurses, and a social worker	Women who had sought abortion; priests; doctors; gynaecologists; nurses; social workers	Unclear but in private places	Not clear	Low
Ouedraogo et al. (2020)^90^	Burkina Faso	Qualitative: ethnography - participant observation and in-depth interviews	52 (39 patients, 13 health care providers)	Women seeking post-abortion care; abortion providers in the study facilities.	Clinics/hospitals; Urban	Participant observation; semi-structured questionnaires	High
Akin et al. (2005)^91^	Turkey	Partially randomized controlled trial	470	Women aged 18–49; enrolled over an eight-month period if they had an intrauterine pregnancy up to 56 days last normal period	Clinic; Urban	Medical and home records	High
Ekanem et al. (2009)^92^	Nigeria	Cross-sectional study	492	Patients admitted to the ward for management of induced abortion and had complete information at 6-weeks follow up.	Hospital; Urban	Structured interviews; patients' case files, ward, and operation theatre records	Low
Palak et al. (2019)^93^	India	Cross-sectional study	6876	Women aged 15–49 years, irrespective of their marital status; history of abortion in five years preceding the survey	Household	Survey questionnaires	High
Rachana et al. (2007)^94^	Nepal	Cross-sectional study	100	Patients attending hospital for comprehensive abortion services	Hospital; Urban	Structured questionnaires; patient files	Low
Biney et al. (2017)^95^	Ghana	Cross-sectional study	552	Women aged 15–49 years; terminated pregnancy and gave reasons for the abortion in the 5 years preceding the survey	Households; countrywide	Structured Women's questionnaire	High
Pilecco et al. (2015)^96^	Brazil	Qualitative reconstruction of quantitative data	18	Women living with HIV aged between 18 and 49 years old and seen in public health services in Porto Alegre, Brazil who reported an abortion after the diagnosis of HIV	Clinic - HIV positive women from specialised services; HIV negative women from primary health services	Reconstruction of quantitative information collected in the primary study	High
Tamang et al. (2012)^97^	Nepal	Cross-sectional study	1041	Clinically verified uterine gestation of 63 days or less; had undergone abortions at study clinics	Clinic	Structured questionnaires	High
Byrne et al. (2021)^98^	Nigeria	Cross-sectional, national survey	1144	Women who had reported abortion in the national survey	National	Survey Questionnaires	High

IDIs – In-depth interviews, KIIs- key informant interviews, FGDs – focus group discussions.

### Abortion decision-making trajectories

**Figure 3:**
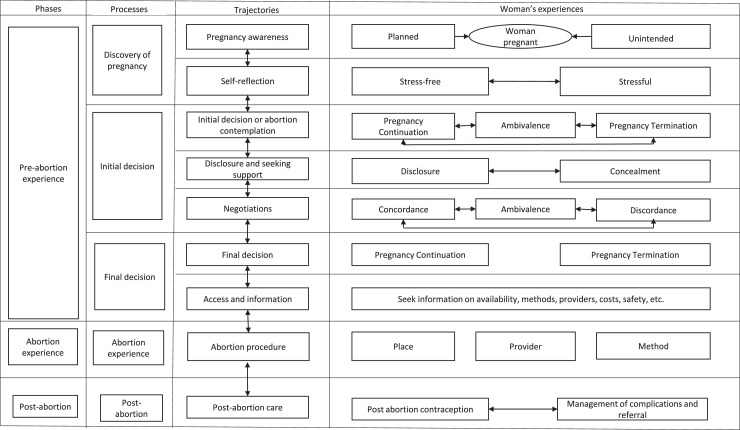
Abortion decision-making trajectories.

We identified nine abortion decision-making trajectories from the synthesis of included studies: pregnancy recognition or awareness, self-reflection, abortion contemplation or initial decision, disclosure and seeking support, negotiations, making a final decision, accessing abortion services, and seeking information, the abortion procedure, abortion outcome, and post-abortion experience and care (Figure 3). These nine trajectories involve complex permutations of cyclical and repetitive steps that influence the temporal relationship from the time the woman discovers the pregnancy to the post-abortion period.

The abortion decision-making trajectories span over three phases: the pre-abortion phase, the actual abortion experience and the post-abortion experience.

(i) *The pre-abortion abortion phase*

The abortion decision-making process starts with the discovery of a pregnancy which may be expected or unexpected, planned or unplanned.^8^, ^26^, ^27^, ^28^, ^29^, ^30^, ^31^, ^32^, ^33^, ^34^, ^35^, ^36^ This is followed by self-reflection where the woman weighs the risks of continuing or terminating the pregnancy which may be stressful or stress-free for different women.^8^^,^^9^^,^^12^^,^^35^, ^36^, ^37^, ^38^, ^39^ The woman then contemplates whether to terminate or continue the pregnancy which may involve periods of ambivalence, weighing the pros and cons of each choice.^8^, ^12^, ^26^, ^30^, ^31^, ^32^, ^33^, ^35^, ^37^, ^39^, ^40^, ^41^, ^42^ The woman may then disclose or conceal the pregnancy or her initial decision to her close networks including partner, parents, siblings, teachers and other close friends depending upon the level of trust, perceived reaction to the woman's decision and expected support.^8^, ^12^, ^31^, ^33^, ^35^, ^36^, ^38^, ^39^, ^43^, ^44^, ^45^, ^48^

Upon pregnancy disclosure, the woman may receive support (concordance), opposition (discordance) or indecision (ambivalence) to her initial abortion decision.^8^, ^26^, ^31^, ^33^, ^35^, ^36^, ^39^, ^45^, ^46^ The woman and the actors to whom the abortion decision has been disclosed to may then enter into a stage of negotiation in cases of discordance and indecision where the woman tries to convince her social networks to agree to her initial decision.^7^^,^^8^^,^^26^^,^^31^^,^^33^^,^^44^ The negotiation process in concordant cases can hasten the decision-making process, but in discordant and ambivalent cases, it can complicate and lengthen the decision-making process*.*^7^^,^^8^^,^^12^^,^^31^^,^^36^

The final abortion decision may be made solely or primarily by the woman herself, jointly with her partner or others, or by others without or with minimal involvement of the woman.^7^, ^8^, ^10^, ^12^, ^26^, ^27^, ^29^, ^30^, ^32^, ^33^, ^34^, ^35^, ^36^, ^37^, ^38^, ^39^, ^40^, ^41^, ^42^, ^44^, ^46^, ^47^, ^48^, ^49^, ^50^, ^51^, ^52^, ^53^, ^54^, ^55^, ^56^, ^57^, ^58^, ^59^, ^60^, ^61^, ^62^, ^63^, ^64^, ^65^, ^66^, ^67^, ^68^, ^69^, ^70^, ^74^ However, even cases in which the majority of the decisions are made by woman, male partners and/or parents still influenced her decision.^7^^,^^35^^,^^39^^,^^44^^,^^74^ Thus, decisions made by woman may either be passive in which the woman simply agrees to the decision imposed onto her by others^8^, ^30^, ^32^, ^35^, ^37^, ^38^, ^39^, ^44^, ^48^, ^51^, ^54^, ^57^, ^60^, ^64^, ^65^, ^66^, ^70^, ^74^ or active in which she actively participates in the decision-making processes.^29^, ^32^, ^36^, ^37^, ^39^, ^40^, ^42^, ^47^, ^53^, ^62^, ^64^, ^65^, ^66^, ^69^ Where the woman is excluded or plays a very limited role in decision-making, threats, coercion, violence, or trickery may be used by their partners and/or parents to coerce her accept their decision^8^^,^^12^^,^^32^^,^^33^^,^^37^^,^^41^^,^^44^^,^^55^^,^^56^ reflecting varying autonomy in women's abortion decision-making.^33^^,^^52^

Once the final decision has been made, women and their social networks such as parents or partners may consult multiple sources to obtain information about availability, affordability, safety, or accessibility to abortion services. Various sources of information include skilled abortion providers, chemists or unskilled abortion providers, intermediaries or brokers, partners, social networks such as friends, parents, teachers, and relatives, the media and internet or school.^12^^,^^26^, ^27^, ^28^, ^29^^,^^32^^,^^34^, ^35^, ^36^^,^^38^^,^^45^^,^^48^, ^49^, ^50^^,^^53^^,^^54^^,^^57^^,^^61^^,^^63^^,^^68^^,^^74^, ^75^, ^76^

(ii) *The abortion phase*

This phase includes the woman's encounter with the abortion providers using safe or unsafe abortion methods in places that may be safe or unsafe biomedically. The abortion procedure includes the actual pregnancy termination which may be carried out clandestinely or overtly, and may involve safe or unsafe procedures or methods,^6^, ^10^, ^12^, ^26^, ^27^, ^28^, ^29^, ^31^, ^32^, ^34^, ^35^, ^36^, ^37^, ^39^, ^41^, ^43^, ^44^, ^45^, ^47^, ^48^, ^49^, ^50^, ^53^, ^55^, ^56^, ^57^, ^58^, ^59^, ^60^, ^61^, ^62^, ^63^, ^64^, ^66^, ^70^, ^74^, ^77^, ^78^, ^79^, ^80^, ^81^, ^82^, ^98^ place of abortion^6^, ^7^, ^26^, ^29^, ^34^, ^35^, ^36^, ^39^, ^41^, ^43^, ^44^, ^45^, ^47^, ^49^, ^50^, ^53^, ^56^, ^60^, ^61^, ^62^, ^63^, ^66^, ^70^, ^72^, ^74^, ^77^, ^82^, ^83^, ^98^ and/or provider.^26^, ^27^, ^28^^,^^34^, ^35^, ^36^, ^37^^,^^39^^,^^43^, ^44^, ^45^^,^^47^^,^^49^^,^^50^^,^^53^, ^54^, ^55^^,^^60^, ^61^, ^62^, ^63^, ^64^^,^^66^^,^^74^^,^^77^^,^^79^^,^^81^^,^^83^^,^^92^^,^^98^ The process may also be legal or illegal as per the country's law. The abortion procedure may result in a safe or unsafe abortion. Safety is understood by women to include not only medical safety (outcome) but also social safety (confidentiality, privacy, secrecy, and reputational safeguarding of the woman and/or her family), legal safety (protection from the law), and financial safety (protection from catastrophic expenditures).^6^, ^9^, ^12^, ^28^, ^32^, ^35^, ^36^, ^37^, ^38^, ^40^, ^41^, ^44^, ^45^, ^47^, ^48^, ^51^, ^53^, ^54^, ^56^, ^58^, ^59^, ^60^, ^61^, ^62^, ^63^, ^75^, ^78^, ^79^, ^81^, ^83^, ^84^, ^85^, ^98^

(iii) *The post-abortion phase*

Depending on the outcome of the abortion procedure, the woman may develop complications which are managed at different referral points including the hospital and by skilled trained abortion providers such as gynaecologists.^6^^,^^26^^,^^27^^,^^32^^,^^33^^,^^36^^,^^38^^,^^40^^,^^44^^,^^45^^,^^47^^,^^49^^,^^55^^,^^60^, ^61^, ^62^^,^^74^^,^^81^, ^82^, ^83^, ^84^ Adolescents, women living with HIV, and commercial sex workers face additional barriers to access to post abortion care including requirements for consent from parents or partners, or stigma.^36^^,^^39^^,^^44^^,^^61^^,^^62^ In only two of the included studies were women given post-abortion family planning.^38^^,^^48^

The details of the trajectories and women's experiences, supported by accompanying quotes, are given in supplementary material.

### Quantitative findings on final decision-making

**Figure 4:**
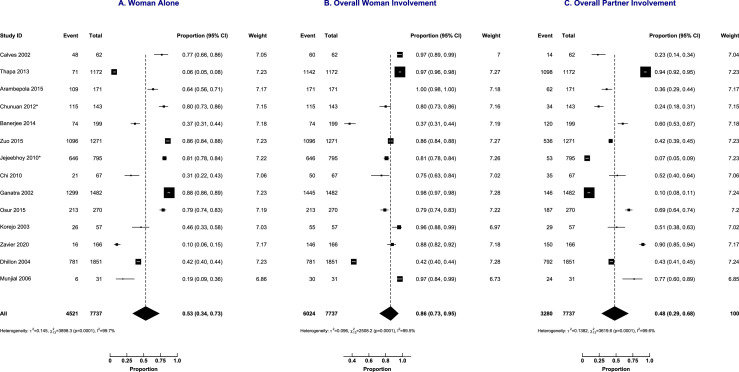
Abortion decision-making involvement by the woman alone, overall woman's involvement and overall male partner's involvement.

A meta-analysis of data from 14 studies of 7737 women showed that the proportion of women's primary or sole involvement in the decision-making was 0.53 (95% CI: 0.34 to 0.73; prediction intervals (PI): 0.20 to 0.99, I^2^ = 99.7%). The overall women's involvement in the abortion decision-making was 0.86 (95% CI: 0.73 to 0. 95; PI: 0.38 to 1.00, I^2^ = 99.5%) and overall partner involvement was 0.48 (95% CI: 0.29 to 0.68; 95% PI: 0.13 to 0.98, I^2^ = 99.6%) as shown in Figure 4. Hence, in 14% and 52% of the cases, women and male partners respectively were excluded from the abortion decision-making process.

**Figure 5:**
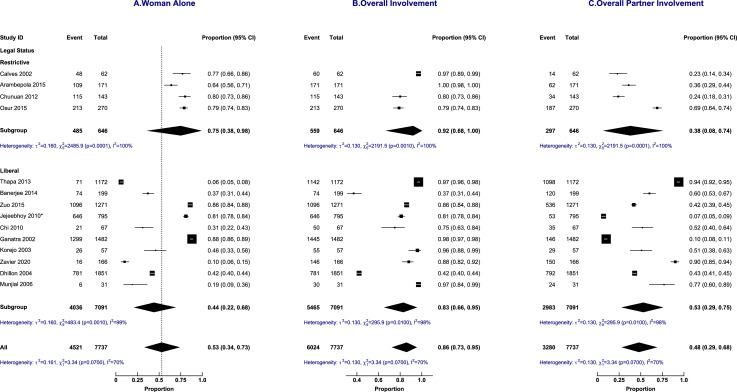
Subgroup analysis by a country's abortion laws of abortion decision-making involvement by the woman alone, overall woman's involvement and overall male partner's involvement.

**Figure 6:**
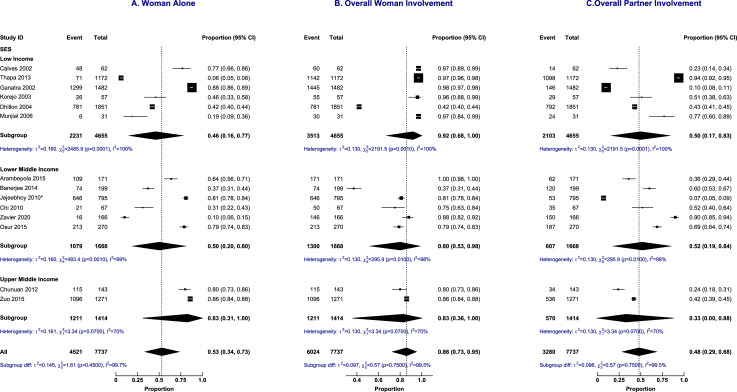
Subgroup analysis by a country's income status of abortion decision-making involvement by the woman alone, overall woman's involvement and overall male partner's involvement. (Footnote for Figure 6: SES – country's socioeconomic status).

**Figure 7:**
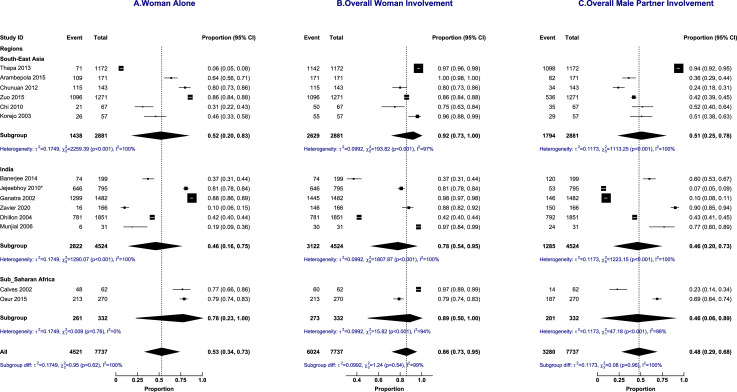
Subgroup analysis by geographical regions of abortion decision-making involvement by the woman alone, overall woman's involvement and overall male partner's involvement.

Sub-group analysis did not explain the heterogeneity in the decision-making trajectories as shown in Figure 5 (abortion laws), Figure 6 (income status), and Figure 7 (geographical regions). Sensitivity analyses did not show any difference in the proportions of decision-making based on the quality of included papers and study setting (results in supplementary material).

### Determinants of abortion trajectories

We synthesised three major themes: (a) autonomy in decision-making, (b) access, and (c) choices with eight sub-themes.

(a) Women's autonomy in decision-making
  (i) Women's autonomy in decision-making: empowerment and coercion

Women exercised varying degrees of involvement in the abortion decision-making processes including the decision to continue or terminate the pregnancy and the choice for the place, method, and provider for abortion.^26^, ^30^, ^31^, ^33^, ^36^, ^45^, ^48^, ^49^, ^50^, ^64^, ^65^, ^66^, ^67^ Women passively participated in the abortion decisions by being bystanders^8^, ^30^, ^32^, ^37^, ^38^, ^39^, ^44^, ^48^, ^51^, ^54^, ^57^, ^60^, ^64^, ^65^, ^66^, ^67^, ^70^, ^74^ or actively by being involved directly in the decision-making processes.^29^, ^30^, ^32^, ^34^, ^37^, ^40^, ^41^, ^42^, ^46^, ^47^, ^53^, ^59^, ^64^, ^65^, ^66^, ^69^, ^70^

*“I talked to him, and he said okay we are going to have an abortion and I accepted”. (22 years). “They decided while I was at school. If (it) was my decision I would keep it because I wanted it”. (female, 18 years old, Mozambique)*.^41^

In cases where women were excluded or played very limited roles, threats, coercion, violence, or trickery were often employed by partners or parents to get the woman to terminate the pregnancy.^8^^,^^12^^,^^32^^,^^33^^,^^37^^,^^41^^,^^55^^,^^56^

*“My boyfriend started threatening me, he sent his friends to talk to me, they cajoled me, threatened me, and tried other things. I didn't change my position. Then, his friends stopped talking to me, even my friend stopped talking to me.” (female, 23 years old, Burkina Faso).*^8^

  (ii) Role and influence of male partners and other social networks in decision-making

Male partners and other social networks (friends, parents, relatives, teachers, abortion providers) played important roles in the abortion decision-making processes including the decision to terminate or continue the pregnancy, and choice for the place, the method or provider for the abortion.^6^, ^7^, ^8^, ^32^, ^35^, ^36^, ^37^, ^39^, ^40^, ^41^, ^49^, ^51^, ^52^, ^57^, ^58^, ^59^, ^62^, ^63^, ^74^, ^76^, ^79^, ^80^, ^81^, ^82^, ^84^, ^87^, ^88^, ^89^, ^98^ Men either supported or opposed women's actions and directly influenced the abortion decision-making trajectories.

*“I wanted to keep it but he said, "Why are you so eager to keep it? It must be someone else's. If it is mine, you will do as I say. After that what could I do?" (female, 19 years old, India).*^37^

Concordant partner involvement in abortion was associated with seeking and obtaining safe abortion.^7^

*"He asked if I could keep it and if I could face that, and then we talked about it. Then we decided it's better [if] we don't bring problems to the [family members paying for our education] ... He came to see me so that we [could] talk about it and decide what we were going to do. We talked about it for like two weeks, then we decided to have a termination.” (female, 19 years old, Zambia).*^7^

(b) Access
  (i) Abortion laws and policies

Restrictive laws compelled women to seek clandestine and backdoor abortion services which left them perilous to any post-abortion complications for which the providers will not be held accountable.^29^^,^^36^^,^^45^^,^ ^50^^,^^52^^,^^61^^,^^84^

*“[Abortion] services are not legal. If you go to a health centre for abortion, they will tell you: "We don't do that". And if they have to do it for you, it's just back door. And the blame is on you. They will give you the medicine and tell you to go and sort yourself, or they will do it for you, and in case of any risk, they will say they were not part of it. And also in the health facility, they don't offer safe abortion.” (female, unknown age, Kenya).*^50^

Abortion laws affected access to information on abortion services including availability of prescription or type of facilities offering services.^12^, ^28^, ^36^, ^49^, ^50^, ^53^, ^54^, ^56^, ^63^, ^79^, ^82^, ^85^

*“The hardest part was getting the prescription ... in the end I got it through a friend of a friend who was a doctor ... but before that I made several appointments with gynaecologists trying to find a friendly doctor who would give me a prescription.” (female, 25 years old, Argentina).*^82^

Even where abortion was legal, limited knowledge of existing abortion laws and policies by abortion providers and seekers hindered access to safe and prompt abortion services.^6^^,^^10^^,^^32^^,^^51^^,^^56^^,^^84^

*"Abortion is legal ... most women don't know. I had information from a druggist who is a nurse.” [female, 27 years old, Ghana].*^84^*NB: Abortion is not legal in Ghana, but the abortion policy allows abortion in some cases.*

In addition, policies that placed onerous requirements for abortion services such as waiting periods, sign-offs or consent forms, and varying limits on gestational age compelled women to seek clandestine abortions.^6^^,^^37^^,^^41^

*“I heard that to induce abortion at the hospital it is necessary for an adult to sign a consent form. I was afraid because I did not know who could accompany me. Because at that time I only wanted to hide it from others.”(female, 22 years old, Mozambique).*^41^

  (ii) Knowledge and information

Knowledge and access to correct information regarding the abortion procedures and recognition of pregnancy were important in the decision to either keep the pregnancy or to terminate.^6^, ^10^, ^12^, ^32^, ^33^, ^41^, ^45^, ^52^, ^53^, ^58^, ^59^, ^61^, ^74^, ^76^, ^78^ Lack of information led to delays in seeking abortion services^78^ with many women being charged higher fees due to advanced pregnancy and clandestine abortion.^52^^,^^58^

*"I did not have a clear idea about abortion ... and besides that, [I had seen] awful videos about abortion ... in school. I thought it would be like I had seen, where the baby is torn apart". [16-year-old, terminated pregnancy].*^33^

  (iii) Affordability

Women and other actors considered cost as an important factor when choosing the method, place or provider for abortion services.^6^, ^27^, ^28^, ^36^, ^37^, ^38^, ^40^, ^41^, ^43^, ^45^, ^48^, ^50^, ^51^, ^61^, ^74^, ^75^, ^77^, ^78^, ^80^, ^81^, ^83^, ^85^, ^86^ In terms of cost, women preferred medical abortion (misoprostol) over surgical abortion as it considered cheaper and more affordable.^28^^,^^40^^,^^48^^,^^51^^,^^77^^,^^83^^,^^86^ For clandestine abortions obtained cheaply which resulted in complications,^27^^,^^37^^,^^40^^,^^43^^,^^50^^,^^83^ post abortion care resulted in extra costs.^6^^,^^78^^,^^86^ Moreover, clandestine abortions resulting from advanced gestational age were more expensive than those at lower gestational age.^80^^,^^86^ However, in contexts where public facilities offered free abortion services, other indirect costs such as distance,^37^^,^^40^ need for hospital fee^81^ or illicit fees^43^ were reported to be prohibitive for women seeking abortion services. However, costs did not preclude women's need for abortion.^38^^,^^50^^,^^51^

*“I did not consider things to do with price. I just wanted it to be terminated.” (female, 29 years old, Kenya)*^38^

(c) Choices
  (i) Women's perceptions of safe abortion

While women acknowledged the need for medical safety in seeking abortion, to them abortion safety encompassed social, legal, reputational, relationship and economic security.^6^, ^9^, ^12^, ^28^, ^32^, ^35^, ^37^, ^38^, ^40^, ^41^, ^45^, ^47^, ^48^, ^51^, ^56^, ^58^, ^59^, ^60^, ^61^, ^62^, ^63^, ^75^, ^78^, ^79^, ^81^, ^83^, ^84^, ^85^, ^98^

*"You may have the best doctors and equipment there, but it is not safe because they will keep your file and everybody will know what you came to do...they also make you pay heavily even when you say you don't have money. That's why those places are not safe for abortion." (female, 29 years old, Kenya).*^79^

Social safety influenced the decision to terminate pregnancy, especially where a pregnancy was associated with stigma,^34^^,^^36^^,^^37^^,^^39^^,^^59^^,^^61^^,^^63^^,^^78^ and to whom it was disclosed.^12^^,^^34^^,^^36^^,^^39^^,^^44^^,^^59^^,^^60^ Social safety also influenced choosing medical abortion or telemedicine as it allowed women to terminate the pregnancy privately and secretly.^29^, ^32^, ^35^, ^36^, ^40^, ^56^, ^58^, ^63^, ^74^, ^75^, ^79^, ^83^

Adolescent and unmarried girls sought abortion far away from home and from providers they did not know,^48^^,^^59^ others preferred pharmacies or community shops over hospitals because pharmacies were thought to keep women's requests for abortion drugs a secret compared to doctors at hospitals.^28^^,^^79^

Furthermore, some hospitals were known to involve long waiting times, required signing consent forms and some did not provide privacy which risked exposing women.^28^^,^^41^^,^^75^^,^^85^ Women also shunned separate and isolated abortion clinics as it made women going there for abortion easily identifiable.^75^ To minimise risk of exposure, some women sent male friends or their boyfriends to buy misoprostol on their behalf,^28^ and repeat abortions were sought from different providers for fear that *“they will say this girl has come again”*.^51^ Traditional herbalists were reputed for their secrecy and confidentiality^79^ while physicians permitted by law to provide abortion under certain conditions in restrictive settings were shunned because they did not need to keep the abortion a secret unlike backstreet abortionists.^60^^,^^62^ They also allowed women to self-induce thereby protecting confidentiality and their “image”.^6^^,^^37^^,^^41^^,^^47^^,^^59^^,^^78^^,^^84^

  (ii) Availability of preferred abortion methods and providers

In choosing abortion services, women considered access and availability as key determinants.^6^, ^9^, ^28^, ^29^, ^32^, ^35^, ^36^, ^37^, ^38^, ^40^, ^43^, ^45^, ^48^, ^51^, ^52^, ^54^, ^56^, ^58^, ^59^, ^62^, ^74^, ^75^, ^76^, ^77^, ^78^, ^80^, ^82^, ^83^, ^85^, ^88^ These included the proximity of services including the number of providers and distance to facilities offering abortion,^37^^,^^40^^,^^54^^,^^58^^,^^59^^,^^78^^,^^88^^,^^98^ availability of services and providers and quality of services.^32^^,^^38^^,^^43^^,^^48^^,^^58^^,^^76^^,^^85^ Other key considerations included the convenience and comfort at the facility including presence of female abortion providers,^40^^,^^48^^,^^58^^,^^62^^,^^63^ reputation of the facility,^29^^,^^32^^,^^37^^,^^43^^,^^59^^,^^75^^,^^76^^,^^82^^,^^83^^,^^85^ and ease of use of the available services.^9^^,^^37^^,^^38^^,^^76^^,^^85^

*“When my pregnancy was confirmed I knew I had to get abortion. I was considering visiting a doctor, so I asked my friend about it. She said I could do it myself by medication and did not need to visit a doctor. She said MA kits are easily available at medical stores and I don't even need a prescription for it. So, I went and bought MA kit from the medical store.” (female, 23 years old, India).*^32^

  (iii) Attitudes of abortion providers

Women reported that trust and confidence in the healthcare providers influenced their decision to go to them for abortion services.^37^^,^^48^^,^^62^^,^^63^^,^^76^^,^^85^^,^^88^^,^^89^^,^^98^

*“[The doctor] told me at the beginning that she believed it was a woman's choice to have children or not, that she was actually one of the doctors who defended women before the law because she performed abortions. That made me feel very good.” (female, 29 years old, Mexico)*.^89^

However, in most cases, the women reported that the health workers were rude, judgemental, abusive, and some tried to impose their own views on the women regarding the abortion decision and made them feel “guilty” which compelled many women to seek clandestine abortion services from elsewhere.^28^, ^29^, ^36^, ^37^, ^38^, ^44^, ^50^, ^58^, ^61^, ^62^, ^84^

*“They do insult patients. You can go to the hospital and then the doctors start talking ill about you, so this discourages you so much, and you decide to leave.” (female, unknown age, Kenya).*^50^

Judgemental attitude, conscientious objection and outright rejection to provide abortion services on moral, social or religious views,^29^^,^^51^^,^^76^^,^^78^^,^^84^ extortion when providing illegal abortion or taking advantage of women with stigmatised abortion^6^^,^^36^^,^^43^^,^^58^^,^^89^ and lack of provider's skills or training in providing abortion services^78^^,^^84^ compelled women to seek abortion services away from the formal abortion providers.

*“Even for me it [rude treatment by health-care providers] is the reason why I stayed away from the hospital.” (female, 28 years old, Ghana)*^84^

By contrast, women preferred traditional herbalists and private practitioners and some private facilities where providers were known to be supportive and understanding.^27^^,^^29^^,^^33^^,^^57^^,^^60^^,^^76^^,^^85^^,^^89^

### Key recommendations

We synthesised eight key recommendations from the included studies:

(i) Empower women to make independent, autonomous decisions.
(ii) Involve, where appropriate, male partners in abortion discourses.
(iii) Legalise abortions and accompany this with pragmatic policies.
(iv) Improve access to information and knowledge.
(v) Provide low cost or free abortion services as permitted by law.
(vi) Broaden the definition of safe abortion to include social safety.
(vii) Ensure availability of acceptable and preferred abortion methods.
(viii) Train and build capacity for health workers to improve their interpersonal and communication skills and to improve their attitude towards women seeking abortion services in legally liberal settings.

A summary of the GRADE-CERQUal assessing the confidence in the evidence related to each recommendation is provided in Table 2. There was high confidence in the evidence used to develop seven of the seven major recommendations with the evidence relating to abortion laws and policies having moderate confidence (recommendation v). There were moderate or minor methodological limitations for all the major determinants, but no minor or major concerns about the evidence for coherence, adequacy, or relevance for the seven recommendations.

**Table 2:** GRADE-CERQUal summary of findings and recommendations.

Summary of findings and recommendations	ID numbers of studies contributing to the review finding	CERQUAL assessment of confidence in the evidence	Explanation of CERQUal assessment
1. Empower women to make independent, autonomous decisions: This may include providing socioeconomic opportunities to offset overreliance on their partners for economic support in order to utilise abortion services	^8^^,^^12^^,^^26^^,^^29^, ^30^, ^31^, ^32^, ^33^, ^34^^,^^36^, ^37^, ^38^, ^39^, ^40^, ^41^, ^42^^,^^44^, ^45^, ^46^, ^47^, ^48^, ^49^, ^50^, ^51^^,^^53^, ^54^, ^55^, ^56^, ^57^^,^^59^^,^^60^^,^^64^, ^65^, ^66^, ^67^^,^^69^^,^^70^^,^^74^	High confidence	Thirty-seven studies with moderate methodological limitations, no or minor concerns about coherence, adequacy or relevance (all studies from LMICs)
2. Involve, where appropriate, male partners in abortion discourses: Men play important roles in abortion decision-making processes. Support of male partners in the decision-making processes diminishes clandestine and unsafe abortions.	6, 7, 8^,^^32^^,^^35^, ^36^, ^37^^,^^39^, ^40^, ^41^^,^^45^^,^^49^^,^^51^^,^^52^^,^^57^, ^58^, ^59^^,^^62^^,^^63^^,^^74^^,^^76^^,^^79^, ^80^, ^81^, ^82^^,^^84^^,^^87^, ^88^, ^89^^,^^98^	High confidence	Thirty-one studies with moderate methodological limitations, no or minor concerns about coherence, adequacy or relevance (all studies from LMICs)
3. Provide policy for legalising and decriminalisation of abortion and accompany this with pragmatic policies: Removing restrictions on abortion does not necessarily results in all abortions being safe. This must be accompanied by changes in policies including expanding the scope of the facilities, providers, and conditions for obtaining safe abortion. Onerous additional requirements such as parental or partner consent, paperwork or permissions lead to delays in obtaining abortion services and often lead to unsafe abortion trajectories. At best, these must be minimised or eliminated altogether. Policies to address conscientious objection (such as timely referral) need to be enacted.	^6^^,^^10^^,^^12^^,^^28^^,^^29^^,^^32^^,^^36^^,^^37^^,^^41^^,^^45^^,^^49^, ^50^, ^51^, ^52^, ^53^, ^54^^,^^56^^,^^61^^,^^63^^,^^79^^,^^82^^,^^84^^,^^85^	Moderate confidence	Twenty-three studies with moderate methodological limitations, moderate concerns about adequacy and no or minor concerns about coherence or relevance (all studies from LMICs).
4. Improve access to information and knowledge: Where abortion is recently legalised, deliberate efforts must be made to ensure that information on the legality of abortion and any requirements/limitations including eligibility, places and providers for abortion are widely disseminated.	^6^^,^^10^^,^^12^^,^^32^^,^^33^^,^^41^^,^^45^^,^^52^^,^^53^^,^^58^^,^^59^^,^^61^^,^^74^^,^^76^^,^^78^	High confidence	Fifteen studies with moderate methodological limitations, no or minor concerns about coherence, adequacy or relevance (all studies from LMICs)
5. Provide low-cost or free abortion services as permitted by law: In communities in which abortion is legal, they should be made free for all women. Adolescent women are at a particular risk of following abortion trajectories that result in unsafe abortion due to unaffordable costs in obtaining abortion services.	^6^^,^^27^^,^^28^^,^^36^, ^37^, ^38^^,^^40^^,^^41^^,^^43^^,^^45^^,^^48^^,^^50^^,^^51^^,^^61^^,^^74^^,^^75^^,^^77^^,^^78^^,^^80^^,^^81^^,^^83^^,^^85^^,^^86^^,^^98^	High confidence	Twenty-four studies with moderate methodological limitations, no or minor concerns about coherence, adequacy or relevance (all studies from LMICs)
6. Broaden the definition of safe abortion to include social safety: There is a need to ensure abortion services are provided in a socially safe environment that ensures confidentiality, privacy and secrecy for women obtaining abortion. There is need for integration of abortion services into the broader sexual and reproductive health services in facilities providing them.	^6^^,^^9^^,^^12^^,^^28^^,^^32^^,^^35^, ^36^, ^37^, ^38^, ^39^, ^40^, ^41^^,^^44^^,^^45^^,^^47^^,^^48^^,^^51^^,^^56^^,^^58^, ^59^, ^60^, ^61^, ^62^, ^63^^,^^74^^,^^75^^,^^78^^,^^79^^,^^81^^,^^83^, ^84^, ^85^^,^^98^	High confidence	Thirty-four studies with moderate methodological limitations, no or minor concerns about coherence, adequacy or relevance (all studies from LMICs)
7. Ensure availability of acceptable methods for abortion services: Different women preferred different methods and in situations where abortion is legally provided, all the safe and effective methods must be available to allow women to make informed choices.	^6^^,^^9^^,^^28^^,^^29^^,^^32^^,^^35^, ^36^, ^37^, ^38^^,^^40^^,^^43^^,^^45^^,^^48^^,^^51^^,^^52^^,^^54^^,^^56^^,^^58^^,^^59^^,^^62^^,^^63^^,^^74^, ^75^, ^76^, ^77^, ^78^^,^^80^^,^^82^^,^^83^^,^^85^^,^^88^^,^^98^	High confidence	Thirty-two studies with moderate methodological limitations, no or minor concerns about coherence, adequacy or relevance (all studies from LMICs)
8. Train and build capacity for health workers to improve their interpersonal and communication skills and to improve their attitude towards women seeking abortion services in legally liberal settings: In addition to expanding the scope of abortion providers, they should have refresher trainings on any newer methodological advances in abortion services. Additionally, continued training in interpersonal and communications skills of providers are important to address negative and judgemental attitude towards women seeking abortion services.	^6^^,^^27^, ^28^, ^29^^,^^33^^,^^36^, ^37^, ^38^^,^^43^^,^^44^^,^^48^^,^^50^^,^^51^^,^^57^^,^^58^^,^^60^, ^61^, ^62^, ^63^^,^^76^^,^^78^^,^^84^^,^^85^^,^^88^^,^^89^^,^^98^	High confidence	Twenty-seven studies with moderate methodological limitations, no or minor concerns about coherence, adequacy or relevance (all studies from LMICs)

## Discussion

This systematic review mapped out nine interlinked abortion decision-making trajectories that highlight the complexity and uncertainty of women's experiences with the abortion decision-making process. The main determinants of abortion trajectories include autonomy, access and choice. The meta-analysis further demonstrated the complexity and heterogeneity of abortion decision-making with overall partner involvement approximating women's involvement in final abortion decision-making.

These trajectories are similar to “the trajectories of women's abortion-related care” developed by Coast et al.^4^ However, there are important differences with our framework. First, our review focused on LMICs where nearly all unsafe abortion-related morbidity and mortality occurs.^3^ Secondly, we have been able to demonstrate the important role of male partners, parents, other social networks, and abortion providers, making these actors potential targets for interventions focused on safe abortion in LMICs. Finally, we have incorporated post-abortion care and experiences as a continuum of abortion decision-making trajectories, although we acknowledge that not all literature on these topics, which were not the focus of our searches, could be included.

While we have attempted to visualise the abortion decision-making processes for women in LMICs in a simplified model, the women's actual experiences are much more complex than what the framework illustrates. This is because abortion decision-making occurs in a fluid environment, with varying degrees of conflicting rationalisation and emotions.^8^^,^^12^^,^^99^ Added to this unpredictability is the fact that abortion decisions do not occur in isolation but are a product, not only of women's autonomy, but are also influenced by her partner, family, social networks, and the environment in which the abortion takes place including abortion laws and policies and the health system factors.^8^^,^^31^^,^^38^^,^^100^, ^101^, ^102^ While the abortion laws impacted access to abortion services, the abortion decision-making process followed a nearly identical trajectory in both liberal and restrictive settings. This is consistent with the findings of the pooled estimates which showed that abortion decision-making does not vary by abortion laws in LMICs. Other studies have also found that the prevalence of overall and unsafe abortion is similar in LMIC setting irrespective of abortion laws.^103^^,^^104^ We found substantial heterogeneity in the abortion decision-making. Although heterogeneity is ubiquitous in prevalence studies,^23^ in our findings it is likely due to differential measurement or ascertainment of “decision-making” used by the different studies. However, it may also reflect the variability within the abortion decision-making processes even in similar contexts.^37^^,^^71^, ^72^, ^73^

We have shown that women's perceptions of safety including social, economic, and legal safety were as important, if not more important than concerns about the outcome of the procedure such as death in the abortion decision-making. The WHO has historically defined abortion safety in terms of the environment, skills(medical) of the person performing it and the appropriateness of the method.^105^ However, some women held the opposite view in which skilled abortion providers legally permitted to provide abortion, and well-equipped abortion facilities were considered unsafe while traditional and other unskilled abortion providers providing clandestine abortion were considered safe.^28^^,^^60^^,^^79^ We also found that in the context of social, economic and legal safety including minimising unnecessary contact between abortion seekers and abortion providers, medical abortion and telemedicine is an acceptable and preferred abortion method for the majority of women.^32^^,^^40^^,^^56^, ^57^, ^58^^,^^75^^,^^79^^,^^82^ This perception of abortion safety was similar in both legally restrictive and liberal settings, and may be amplified by judgemental communities and abortion providers even where abortion is legal.^106^^,^^107^ Confidentiality remains a core tenet of medical ethics and governs the doctor-patient relationships to ensure mutual trust and confidence in the health system^108^ and our review underscores its importance in the context of providing abortion.

A previous systematic review shown that majority of male partners play important roles in improving access to and utilisation of sexual and reproductive health services such as antenatal care, skilled birth attendance, institutional delivery, postpartum visits, mother's knowledge and recognition of danger signs, and modern contraceptive use.^109^ This review showed that male partners play an important role in abortion decision-making trajectories, including making it easier for women to seek and access safe abortion promptly.^7^

Women's empowerment including autonomous decision-making is associated with mixed results with regards to uptake of sexual and reproductive health services including modern contraceptive, safe abortion, antenatal care, institutional delivery, antenatal and postnatal care.^110^^,^^111^ This may be due to varying degrees to which women can make autonomous decisions with regards to sexual and reproductive health services which may depend on their relationship with their male partners, as demonstrated by the review.

Our review has a number of strengths. First, we conducted an extensive and comprehensive search including multiple databases and the grey literature without language restrictions. Secondly, our review is one of the first to explore abortion decision-making trajectories and their determinants in LMICs where most unsafe abortions occur thus ensuring that factors influencing abortion trajectories in these settings are collated to aid policymakers and programme managers.

The main limitation of the review is related to the methodological limitations of the included studies which may affect the confidence in our findings. Even though the majority of articles were rated high to medium quality, the majority of individual studies had various methodological limitations. For example, only one in five of the mixed methods and qualitative studies included information on reflexivity. Secondly, the studies on abortion decision-making displayed considerable clinical heterogeneity. This was due to operational definitions and measurement of the ’decision’. While some studies provided information on sole decision-maker, others provided information on primary decision-maker, allowing multiple responses for the decision-maker. We could not however do sub-group analysis by operational definitions as some studies were unclear on what definition they had used. Thus, the pooled estimates should be interpreted with caution. Finally, while we have made attempts to delineate the abortion decision-making trajectories through a simplified yet broad conceptual framework, we acknowledge that it may not wholly apply to different circumstances surrounding the abortion decision-making process, such as rape victims, commercial sex workers, refugees, and women living with HIV for which abortion is not medically indicated. However, from the studies included in the review, we observed that rape victims went through the same decision-making trajectories.^7^^,^^37^^,^^75^^,^^78^ Individual women's experiences, vary by context and circumstances, and thus may not be fully captured by this conceptual framework.

In conclusion, our review mapped complex abortion decision-making trajectories and determinants in LMICs leading to the following key recommendations: (i) Empower women to make independent, autonomous decisions; (ii) Engage, where appropriate, male partners in abortion discourses; (iii) Legalise abortions and accompany this with pragmatic policies; (iv) Improve access to information and knowledge; (v) Provide low cost or free abortion services as permitted by law; (vi) Broaden the definition of safe abortion to include social safety; (vii) Ensure availability of acceptable and preferred methods for abortion services; and (viii) Train and build capacity for health workers to improve their interpersonal and communication skills and their attitude towards women seeking abortion services in legally liberal settings.

Employing these could help address unsafe abortion-related morbidity and mortality by targeting specific steps and determinants in the abortion decision-making process. In addition, further research is required on the role of men and other partners in abortion decision-making in LMICs. Although our review focused on women who had abortions, we also noted that many women who wanted to have an abortion did not have it. Future research is needed to delineate the trajectories for these women. Also, the conceptual framework may not aptly capture the abortion decision-making of certain groups of women such as rape victims, commercial sex workers, refugees, adolescents, and women living with HIV for which medical abortion is not indicated. Further research is needed to understand how the broad trajectories framework developed through this review apply to these groups of women.

## Contributors

PL, MN, JK, and CN conceived the idea, planned, and designed the study protocol. PL, IC, JM, and SF did screening, data extraction and quality assessment with input from all authors. CO supported the meta-analysis and MN contributed to interpreting the findings. PL wrote the first draft; IC, JM, SF, CN, JK, CO and MN all edited the draft and provided critical insights. All authors have approved and contributed to the final submitted manuscript. PL, IC, JM, and SF had full access to all the information for the paper and have verified all data included in the review. PL had the final responsibility for the decision to submit for publication.

## Data sharing statement

All the data generated or analysed during this review are included in this published article and its supplementary information files.

## Declaration of interests

The authors declare no conflict of interest.

## Appendix Supplementary materials

Image, application 1
