# Supplementary material for: Abortion decision-making process trajectories and determinants in low- and middle-income countries: A mixed-methods systematic review and meta-analysis
Source: eClinicalMedicine. 2022 Oct 17;54:101694. doi: 10.1016/j.eclinm.2022.101694 (PMC9579809; doi:10.1016/j.eclinm.2022.101694)

*ST 1: Search strategy for Ovid Medline*

| # | Search | 16/02/2021 | 06/06/2022 |
|---|---|---|---|
| 1 | (afghanistan or albania or algeria or american samoa or angola or "antigua and barbuda" or antigua or barbuda or argentina or armenia or armenian or aruba or azerbaijan or bahrain or bangladesh or barbados or republic of belarus or belarus or byelarus or belorussia or byelorussian or belize or british honduras or benin or dahomey or bhutan or bolivia or "bosnia and herzegovina" or bosnia or herzegovina or botswana or bechuanaland or brazil or brasil or bulgaria or burkina faso or burkina fasso or upper volta or burundi or urundi or cabo verde or cape verde or cambodia or kampuchea or khmer republic or cameroon or cameron or cameroun or central african republic or ubangi shari or chad or chile or china or colombia or comoros or comoro islands or iles comores or mayotte or democratic republic of the congo or democratic republic congo or congo or zaire or costa rica or "cote d'ivoire" or "cote d' ivoire" or cote divoire or cote d ivoire or ivory coast or croatia or cuba or cyprus or czech republic or czechoslovakia or djibouti or french somaliland or dominica or dominican republic or ecuador or egypt or united arab republic or el salvador or equatorial guinea or spanish guinea or eritrea or estonia or eswatini or swaziland or ethiopia or fiji or gabon or gabonese republic or gambia or "georgia (republic)" or georgian or ghana or gold coast or gibraltar or greece or grenada or guam or guatemala or guinea or guinea bissau or guyana or british guiana or haiti or hispaniola or honduras or hungary or india or indonesia or timor or iran or iraq or isle of man or jamaica or jordan or kazakhstan or kazakh or kenya or "democratic people's republic of korea" or republic of korea or north korea or south korea or korea or kosovo or kyrgyzstan or kirghizia or kirgizstan or kyrgyz republic or kirghiz or laos or lao pdr or "lao people's democratic republic" or latvia or lebanon or lebanese republic or lesotho or basutoland or liberia or libya or libyan arab jamahiriya or lithuania or macau or macao or republic of north macedonia or macedonia or madagascar or malagasy republic or malawi or nyasaland or malaysia or malay federation or malaya federation or maldives or indian ocean islands or indian ocean or mali or malta or micronesia or federated states of micronesia or kiribati or marshall islands or nauru or northern mariana islands or palau or tuvalu or mauritania or mauritius or mexico or moldova or moldovian or mongolia or montenegro or morocco or ifni or mozambique or portuguese east africa or myanmar or burma or namibia or nepal or netherlands antilles or nicaragua or niger or nigeria or oman or muscat or pakistan or panama or papua new guinea or new guinea or paraguay or peru or philippines or philipines or phillipines or phillippines or poland or "polish people's republic" or portugal or portuguese republic or puerto rico or romania or russia or russian federation or ussr or soviet union or union of soviet socialist republics or rwanda or ruanda or samoa or pacific islands or polynesia or samoan islands or navigator island or navigator islands or "sao tome and principe" or saudi arabia or senegal or serbia or seychelles or sierra leone or slovakia or slovak republic or slovenia or melanesia or solomon island or solomon islands or norfolk island or norfolk islands or somalia or south africa or south sudan or sri lanka or ceylon or "saint kitts and nevis" or "st. kitts and nevis" or saint lucia or "st. lucia" or "saint vincent and the grenadines" or saint vincent or "st. vincent" or grenadines or sudan or suriname or surinam or dutch guiana or netherlands guiana or syria or syrian arab republic or tajikistan or tadjikistan or tadzhikistan or tadzhik or tanzania or tanganyika or thailand or siam or timor leste or east timor or togo or togolese republic or tonga or "trinidad and tobago" or trinidad or tobago or tunisia or turkey or turkmenistan or turkmen or uganda or | 2013224 | 2219677 |

| | | | |
|---|---|---|---|
| | ukraine or uruguay or uzbekistan or uzbek or vanuatu or new hebrides or venezuela or vietnam or viet nam or middle east or west bank or gaza or palestine or yemen or yugoslavia or zambia or zimbabwe or northern rhodesia or global south or africa south of the sahara or sub-saharan africa or subsaharan africa or africa, central or central africa or africa, northern or north africa or northern africa or magreb or maghrib or sahara or africa, southern or southern africa or africa, eastern or east africa or eastern africa or africa, western or west africa or western africa or west indies or indian ocean islands or caribbean or central america or latin america or "south and central america" or south america or asia, central or central asia or asia, northern or north asia or northern asia or asia, southeastern or southeastern asia or south eastern asia or southeast asia or south east asia or asia, western or western asia or europe, eastern or east europe or eastern europe or developing country or developing countries or developing nation? or developing population? or developing world or majority world or less developed countr* or less developed nation? or less developed population? or less developed world or lesser developed countr* or lesser developed nation? or lesser developed population? or lesser developed world or under developed countr* or under developed nation? or under developed population? or under developed world or underdeveloped countr* or underdeveloped nation? or underdeveloped population? or underdeveloped world or middle income countr* or middle income nation? or middle income population? or low income countr* or low income nation? or low income population? or lower income countr* or lower income nation? or lower income population? or underserved countr* or underserved nation? or underserved population? or underserved world or under served countr* or under served nation? or under served population? or under served world or deprived countr* or deprived nation? or deprived population? or deprived world or poor countr* or poor nation? or poor population? or poor world or poorer countr* or poorer nation? or poorer population? or poorer world or developing econom* or less developed econom* or lesser developed econom* or under developed econom* or underdeveloped econom* or middle income econom* or low income econom* or lower income econom* or low gdp or low gnp or low gross domestic or low gross national or lower gdp or lower gnp or lower gross domestic or lower gross national or lmic or lmics or third world or lami countr* or transitional countr* or emerging economies or emerging nation?).ti,ab,sh,kw. | | |
| 2 | exp Abortion, Induced/ | 40511 | 41807 |
| 3 | abortion.mp. | 89438 | 93169 |
| 4 | pregnancy termination.mp. | 2432 | 2578 |
| 5 | 2 or 3 or 4 | 90960 | 94829 |
| 6 | decision making.mp. | 226784 | 253814 |
| 7 | exp Decision Making/ | 207933 | 224714 |
| 8 | 6 or 7 | 333192 | 369831 |
| 9 | 1 and 5 and 8 | 498 | 528 |
| 10 | limit 9 to (humans and yr="2000 -Current") | 323 | 360 |

*ST 2: Articles excluded with reasons for their exclusion*

| Study | Title | Reason for Exclusion |
|---|---|---|
| Biney et al (2017) | Examining the association between motivations for induced abortion and method safety among women in Ghana | Exclusion reason: Wrong outcomes: Reason for abortion |
| Erfani et al (2016) | Levels, trends and correlates of abortion in Tehran, Iran: 2009-2014 | Exclusion reason: Wrong outcomes: Discusses abortion trends |
| Kaye et al (2005) | Reasons, methods used and decision-making for pregnancy termination among adolescents and older women in Mulago Hospital, Uganda | Exclusion reason: Wrong outcomes: Discusses reasons for abortion |
| Bailey et al (2003) | Adolescents' decision-making and attitudes towards abortion in North-East Brazil | Exclusion reason: Wrong outcomes: About attitudes one-year post abortion; |
| Hess et al (2007) | Women's Stories of Abortion in Southern Gabon, Africa | Exclusion reason: Wrong outcomes: Reasons for abortions, methods and post-abortion contraception |
| Gipson et al (2008) | "Having another child would be a life or death situation for her": Understanding pregnancy termination among couples in rural Bangladesh | Exclusion reason: Wrong outcomes: The qualitative findings discuss more of autonomy rather than trajectories |
| Hirz et al (2017) | The role of men in induced abortion decision making in an urban area of the Philippines | Exclusion reason: Wrong outcomes: Reasons for abortions |
| Varga et al (2002) | Pregnancy termination among South African adolescents | Exclusion reason: Wrong patient population: The story on abortion decision-making is a role play |
| Moore et al (2015) | Costs and decision-making regarding abortion and abortion care-seeking in Zambia | Exclusion reason: Wrong outcomes: Cost analysis |
| Zamberlin et al (2012) | Latin American women's experiences with medical abortion in settings where abortion is legally restricted | Exclusion reason: Wrong study design: Literature review; |
| Elul et al (2010) | Assessments of the importance of provider characteristics for abortion care: data from women in Rajasthan, India | Exclusion reason: Wrong patient population: Women did not necessarily have abortions |
| Orner et al (2011) | 'It hurts, but I don't have a choice, I'm not working and I'm sick': decisions and experiences regarding abortion of women living with HIV in Cape Town, South Africa | Exclusion reason: Wrong outcomes: Studies is about challenges women with HIV face in accessing abortion services in South Africa |

| Study | Title | Reason for Exclusion |
|---|---|---|
| Whittaker (2002) | 'The truth of our day by day lives': Abortion decision making in rural Thailand | Exclusion reason: Wrong outcomes: Results are based on vignettes being discussed to explore social norms about abortion and to investigate how people talk about abortion decisions |
| Gipson et al (2011) | Perceptions and practices of illegal abortion among urban young adults in the Philippines: A qualitative study | Exclusion reason: Wrong patient population: This is about an hypothetical pregnancy; |
| Schwandt et al (2013) | Pathways to unsafe abortion in Ghana: The role of male partners, women and health care providers | Exclusion reason: Wrong outcomes: Focus more is on reasons for and experiences of abortion rather than trajectories |
| Capelli et al (2019) | Non-marital pregnancies and unmarried women's search for illegal abortion in Morocco | Exclusion reason: Wrong outcomes: mostly focused on reproductive rights, only briefly mentions a few case studies; Does not directly address the questions |
| Kebede et al (2012) | The tale of the hearts: deciding on abortion in Ethiopia | Exclusion reason: Wrong study design: Case studies |
| Bhilwar et al (2016) | Prevalence of induced abortions and contraceptive use among married women in an urban slum of Delhi, India | Exclusion reason: Wrong outcomes: irrelevant outcomes |
| DaVanzo et al (2014) | Pregnancy Termination in Matlab, Bangladesh: Trends And Correlates of Use of Safer and Less-Safe Methods | Exclusion reason: Wrong outcomes: This is comparing trends and not specific to our question |
| Ray-Bennett et al (2019) | Understanding reproductive health challenges during a flood: Insights from Belkuchi Upazila, Bangladesh | Exclusion reason: Wrong outcomes: About service provision than trajectories, unclear whether the menstrual regulation was for induced abortions or miscarriages |
| Whittaker et al (2002) | Reproducing inequalities: Abortion policy and practice in Thailand | Exclusion reason: Wrong study design: case studies, rest of women excluded |
| Ciren et al (2020) | Pragmatics of everyday life: A qualitative study of induced abortion among Tibetan women in Lhasa | Exclusion reason: Wrong outcomes: Discusses services available and reasons for abortion |
| Mothiba et al (2020) | Experiences leading to the choice of termination of pregnancy amongst teenagers at a regional hospital in Mpumalanga province, South Africa | Exclusion reason: Wrong outcomes: About experiences not pathways or their determinants |
| Sheehy et al (2015) | "We can lose our life for the abortion": exploring the dynamics shaping abortion care in peri-urban Yangon, Myanmar | Exclusion reason: Wrong patient population: The participants are not people who had an abortion; |
| Moseson et al (2020) | Effectiveness of self-managed medication abortion between 13 and 24 weeks gestation: A retrospective review of case | Exclusion reason: Wrong study design: Retrospective review of case records, comparison of methods and linkage to care |

| Study | Title | Reason for Exclusion |
|---|---|---|
| | records from accompaniment groups in Argentina, Chile, and Ecuador | |
| Mumah et al (2020) | Adolescents’ narratives of coping with unintended pregnancy in Nairobi’s informal settlements | Exclusion reason: Wrong outcomes: Reasons for and experiences of abortion |
| Plummer et al (2008) | Aborting and suspending pregnancy in rural Tanzania: an ethnography of young people's beliefs and practices | Exclusion reason: Wrong outcomes: Explored beliefs and perceptions on abortion |
| Scott et al (2018) | A qualitative analysis of decision-making among women with sexual violence-related pregnancies in conflict-affected eastern Democratic Republic of the Congo | Exclusion reason: Wrong outcomes: Focus on outcomes of sexual violence-related pregnancies only |
| Omideyi et al (2011) | Contraceptive practice, unwanted pregnancies and induced abortion in Southwest Nigeria | Exclusion reason: Wrong outcomes: Reasons for and community perceptions of unintended pregnancies and abortion |
| Depiñeres et al (2017) | 'I felt the world crash down on me': Women's experiences being denied legal abortion in Colombia | Exclusion reason: Wrong patient population: This is about women who were denied abortion |
| Webb et al (2000) | Attitudes to 'Kaponya Mafumo': The terminators of pregnancy in urban Zambia | Exclusion reason: Wrong patient population: Participants were school children discussing a hypothetical case |
| Kyilleh et al (2018) | Adolescents' reproductive health knowledge, choices and factors affecting reproductive health choices: a qualitative study in the West Gonja District in Northern region, Ghana | Exclusion reason: Wrong patient population: Discusses issues around abortion by adolescents who had no abortion |
| Gbagbo et al (2015) | Decision-making for induced abortion in the Accra metropolis, Ghana | Exclusion reason: Wrong outcomes: Discusses reasons for abortions, outside the scope of the review |
| Motaghi et al (2013) | Triangular assessment of the etiology of induced abortion in Iran: a qualitative study | Exclusion reason: Wrong outcomes: Reasons for abortion or pregnancy continuation |
| Oduro et al (2014) | "Abortion--it is my own body": women's narratives about influences on their abortion decisions in Ghana | Exclusion reason: Wrong outcomes: Discusses women's autonomy in decision-making |

*ST 3: Abortion decision-making trajectories*

| Process | Trajectory | Description | ID numbers of studies contributing to the findings | Quotations: First or second order |
|---|---|---|---|---|
| Pregnancy awareness | **Pregnancy awareness** | The abortion decision-making trajectory starts with the discovery of pregnancy which may be expected or unexpected. Unexpected pregnancies are likely preceded by "unpreparedness" which may include casual sex or non-use of modern contraceptives. For some women, the pregnancy may be discovered through a pregnancy test or after missing periods. The pregnancy may have been planned by one or both partners. In other cases, the pregnancy may not have been planned at all. | [8,24–34] | *"I found out that I have a son. I had a casual relationship...with a woman I knew...that any time she or I felt like it [having sex], one person would say, "hey, should we see each other" ... and the other person would say, "yes, okay" ... One day she showed up and said, "I'm late. I took a test and I think I'm pregnant." So we went to the doctor, they confirmed it...she was one and a half months along...The doctor talked to us and said everything, "The decision is for both of you to make. If you want to make the decision to not have it, you have 2 - 3 more weeks...You can come to my office or to another office, for them to do an aspiration and in 3 - 4 hours, you'll walk out of my office without a problem ... so talk about it and make the best decision...We talked and we agreed that she wasn't going to have it ... But then she had it. (Male, 32 years old, Mexico)*[28].<br><br>*"When I opened the test and saw that it was positive, I called him, he came at the same time. I was upset, he told me not to worry that everything was going to be fine, that he doesn't earn much money, but what he earns will allow us to manage the pregnancy [...]. When I left him, I went straight to my friends to say that it is positive, that my boyfriend wants me to keep but I want to have an abortion. She agreed that I should have an abortion and she already* |

| Process | Trajectory | Description | ID numbers of studies contributing to the findings | Quotations: First or second order |
|---|---|---|---|---|
| | | | | *knew a good method to have a safe abortion [...] When I explained to him [her boyfriend] that I was going to have an abortion, he didn't want it. [...] But then, as we were talking, he calmed down a bit, he changed his mind."(Female, 23 years old, Burkina Faso)[8].* |
| | **Self-reflection** | While some women may be stress-free during this phase, women may alternate between stressful and stress-free periods. The reaction to a pregnancy may be that of shock, surprise, and stress or ambivalent with periods of stress alternating with stress-free situations. The phase is variable and can take a few hours to weeks or even months. | 8,9,12,33–37 | *"I was still deciding what to do; I was still doubting. So many things run into my mind until I come with the decision to do [---] to [---] to [---] end the pregnancy. At that time [---] [I] even think I lost [---][weight] cause [because of the] stress I have [---]having so many stress [---] losing weight cause of the stress."(Female, 22 years old, Kenya)[12].*<br><br>*Women shared that this time of reflection enables them to decide what they want before the personal decision becomes subject to community perspectives (both supportive and in opposition to the decision(Kenya)[12].* |
| | **Abortion contemplation /initial decision-making** | Following the phase of self-reflection, women may make an initial abortion decision, a decision most often influenced by the environment in which she lives and the perceived reactions of her social networks to the pregnancy. The initial decision may be pregnancy termination, pregnancy continuation or ambivalence | 8,12,24,28–31,33,35,37–40 | *"At first, I decided I wanted to continue... so a few weeks went by and I talked to my grandmother about it.... She helped me and told me to [have the baby]. But when I talked to my mom, things changed. She told me I would lose lots of things, that I would lose the house, school, that they wouldn't give me anything. Then things started to look pretty bleak.... That same night, I got my things together* |

| Process | Trajectory | Description | ID numbers of studies contributing to the findings | Quotations: First or second order |
|---|---|---|---|---|
| | | about the pregnancy but women may go through all these phases iteratively at different time intervals. While the perceived reaction of the social network to the pregnancy may influence the initial abortion decision, adult women often make their decisions independently. Adolescents may defer the initial decision to their parents often due to fear of their parents' reaction to the pregnancy or because they are resigned to the fact that the decision will be made by parents irrespective of their choice. However, even in cases where adolescents may make the initial decision on abortion, parents often override this initial decision if it is mostly at variance with their choice. | | *and said to my dad, "You know what? I'm going to live with [my boyfriend]," but my dad got mad and started to yell. "No, no, no. I don't want you to have [the baby]." [female, 17-year-old, Mexico]*[31]. <br><br> *"At first, I didn't dare talk about it with anyone, even my boyfriend, I didn't want to tell him because I was thinking he would abandon me. I've been thinking for several days, how am I going to do that [tell him]? Finally, I decided that I had to talk to my boyfriend about it since he is also affected (female, 20 years old, Burkina Faso )*[8]. |
| | **Disclosure and seeking support** | Following the initial abortion decision is the phase of disclosure and seeking support. The pregnant woman may disclose to or conceal the pregnancy and the initial decision from her social networks including partner, parents, siblings, teachers, and other close friends. This disclosure and seeking support depend on the level of trust and expected support from the persons to whom the pregnancy or its decision is disclosed. | 8,12,29,31,33,34,36,37,41–43,46 | *"I was doubting what to do and on the other hand afraid of sharing with anybody. I believed if I share it with so many people some people will give me other advice, some will give me this; that's why I ended up sharing with my sister that I'm staying with because I trusted her" (female, 22 years old, Kenya)*[12]. <br><br> *"I did not ask someone for advice because if you ask one they will start talking about it and everybody will know* |

| Process | Trajectory | Description | ID numbers of studies contributing to the findings | Quotations: First or second order |
|---|---|---|---|---|
| | | When the level of trust and support is perceived to be low or non-existent, women may not disclose the pregnancy to their social networks. Some women may only disclose to a person who is expected to make the decision or provide emotional support for the abortion process. For example, adolescents may disclose their pregnancies to their parents who they expect will make decisions on their behalf but may also conceal the pregnancy due to fear or perceived opposition to their initial decision. Disclosure or concealment among adult married women is dependent on level of support from their partner and relationship stability. | | *about it, so I was afraid to talk about it to someone [---] and maybe it will go back to my partner, and I didn't want that to happen." (female, 32 years old, Kenya)*[12]. |
| | **Negotiations/ bargaining** | The woman and the actors to whom the abortion decision has been disclosed to then enter a stage of negotiation in cases of discordance and indecision where the woman tries to convince her social networks to agree to her initial decision. | 7,8,12,24,29,31,44 | *"She is the one who at first told me it was not the baby's fault and that it was me who had been irresponsible.... My friend was basically against the idea of my getting an abortion.... She said, "And what if you do not make it through? What will I do without you?" I said, "Nothing is going to happen. I am going to be fine." [female, 16-year-old, Mexico]*[31]. |
| | **Final decision** | With the completion of the negotiations, the decision-making process may enter | 7,8,10,12,24,25,27,28, 30–40,42–68,72 | *"Then I spoke to my boyfriend and he said, "I am going to do the right thing. We are going to talk to your father." Then* |

| Process | Trajectory | Description | ID numbers of studies contributing to the findings | Quotations: First or second order |
|---|---|---|---|---|
| | | the second stage which begins with the final abortion decision – whether to keep or terminate the pregnancy. The decision may be made jointly with partner/parents, by the woman alone – sometimes without even informing the partner or parents, by the partner alone or primarily, or by others such as parents, in-laws, relatives, friends, and teachers. Even in cases in which the majority of the decisions were made by women, men or parents still influenced their decision. Thus, decisions made by women were either passive in which the woman agreed to the decision imposed on her by others or active in which she actively participated in the decision-making processes. In cases where women were excluded or played a very limited role, threats, coercion, violence, or trickery were often employed by partners or parents to get the woman to terminate the pregnancy reflecting a lack control and power of women in making decisions regarding their own pregnancy. | | *[my father] sent for my boyfriend and asked, "How is it going to be? What are you going to do?" My boyfriend told him, "I am going to be responsible. I am going to do what I have to do." [female, 17-year-old, Mexico]*[31]. <br><br> *"When I had my first pregnancy, I didn't tell my husband about it, so I went to abort it…I thought he was going to get angry because he already told me that he is not ready for pregnancy" [female, 22 years old, Nigeria]*[24]. <br><br> *"I wanted to keep it but he said, "Why are you so eager to keep it? It must be someone else's. If it is mine, you will do as I say. After that what could I do?" (female, 19-year-old, India)*[35]. <br><br> *"It was great, he said, "stop it, what you decided, it's decided. If you want to have it, then you will have it. If you want to have an abortion, I'm with you, don't worry." Still, he is so friendly and supportive of me." [female, 23 years old, Brazil]*[47]. <br><br> *"My dad told me he would disown me if I don't get an abortion" [female, 17 years old, Ghana]*[10]. |

| Process | Trajectory | Description | ID numbers of studies contributing to the findings | Quotations: First or second order |
|---|---|---|---|---|
| | **Access and information** | Once the final decision has been made, women and their social networks such as parents or partners consult multiple sources to obtain information about availability, affordability, safety or accessibility to abortion services. Various sources of information include skilled abortion providers, chemists or unskilled abortion providers, intermediaries or brokers, partners, social networks such as friends, parents, teachers, and relatives, the media and internet or school. | 12,24–27,30,32–34,36,43,46–48,51,52,55,59,61,66,72–74 | *"Once you get pregnant you will know all of those places and the ones who are selling those drugs. You will have their contacts as well as doctor's contacts. You can even find students who are agents for these doctors, and they will direct you to them as they know who are good at this job." (female, 21 years, Tanzania)*[26]*.*<br><br>*"My partner began investigating options on the Internet. He found the government webpage and called the number. They told him where the clinics were, what the requirements were, and that you don't necessarily have to live here in Mexico City, and that in fact a lot of people come from other states."[female, 21 years old, Mexico]*[74]*.*<br><br>*"I knew that I couldn't ask for information about abortion services from people who I am close to and yet keep my pregnancy secret. [...] I did not know even how to raise the issue. But the delala (abortion intermediaries) was there. He approached me while sitting outside the hospital compound. He himself brought up the issue and gave me the information about abortion service providers. Back then I didn't know that he was a delala. He was nice. He promised to find me a provider who gives clean services for a fair price."(female, 19 years old, Ethiopia)*[73]*.* |

| Process | Trajectory | Description | ID numbers of studies contributing to the findings | Quotations: First or second order |
|---|---|---|---|---|
| | **Methods** | Methods used to terminate the pregnancy are either safe or unsafe. Safe methods used include medical abortion (MA) which is preferred by women for its ease and convenience of use, avoidance of painful surgical procedures and because of the control the woman has over the process. Surgical methods including both manual vacuum aspiration (MVA) and dilatation and curettage (D&C) are preferred by some women considering them to be a one-time effective method for terminating the pregnancy with no requirement for visiting the hospital again. The unsafe methods include traditional methods that involve ingestion of over-the-counter drugs such as chloroquine, quinine, and antibiotics, local herbs and roots, insertion of sticks and cassava roots to the cervix by unskilled women or applying blunt manual force to the abdomen. | 6,10,12,24–27,29,30,32–35,37,39,41–43,45–48,51,53–62,64,68,72,75–80,96 | *"Yes. We prefer the [MA] (medical abortion) kit only; it is more trustworthy and we would visit the same store for purchasing the kit in future."(male partner, 31 years, India)*[30].<br><br>*"She asked me to wait and said that the abortion would definitely occur. But my family members started shouting and saying nothing is happening with the tablet and the days (gestational age) are increasing, so I decided to get admitted for a surgical abortion" (female, 35 years old, India)*[55].<br><br>*Unskilled old women who insert sticks or roots of cassava into the cervix carry out another type of traditional method used to provoke unsafe abortion. When left in place, the cassava dilates the cervix and stimulates uterine contractions. Two girls who aborted at home were admitted to the hospital with severe infections after having cassava sticks or roots inserted into their cervices (Zambia)*[75]. |
| | **Place** | Abortion services are obtained at various places including government/public facilities which are considered to be generally safe, cost-effective and have a good reputation. These could be referral | 6,7,24,27,32–34,37,39,41–43,45,47,48,51,54,58– | *"I never knew one could stay in the comfort of your house. I think I really never knew about all that before, all my mind was that abortion must be done in the Health Care for Women International hospital or you start to use all those* |

| Process | Trajectory | Description | ID numbers of studies contributing to the findings | Quotations: First or second order |
|---|---|---|---|---|
| | | centres for management of post abortion complications arising from clandestine and unsafe abortion, private licensed clinics and health facilities which may be run by NGOs and offer subsidised, safe and legal abortion services, private unlicensed clinics that often offer illegal, unsafe, clandestine but low cost abortion services, homes of traditional abortion providers which are preferred for cost-effectiveness, social safety, and ease of access and availability, or at own home, school or neighbour's home which are considered comfortable and permitted for convenient use of medical abortion. Government facilities and private licensed clinics and health facilities often provide safe and legal abortions while all the other abortion services mostly provide unsafe, clandestine and often illegal abortion services. | 61,64,68,70,72,75,80, 81,96 | *local concoctions that you don't even know what the future holds." (female, 29 years old, Nigeria)*[27]*.*<br><br>*"The operating theatre is very big, equipped with powerful lighting, and the table is very big and the sheets are very clean. There was an anaesthetist and I could see so much fancy medical equipment inside the theatre. The doctor was very polite and the theatre staff are equally good and kind to the patients. Doctor "kku rasianna kai" (The doctor has lucky hands). I was asked to change my clothes and they provided me with a neatly pressed hospital gown that I had to wear before I got on the table. The theatre surgeon was very polite and kind. Although I got restless waiting for a long time, still I was pleased with the overall treatment that was given to me. I did not have any complications after getting abortion and sterilisation.(female, unknown age, India)"*[41]*.* |
| | **Providers** | The providers of abortion services include skilled and trained health workers who provided safe abortion services and are often obstetricians/gynaecologists, other medical doctors, nurses or midwives trained in provision of abortion services, health workers not trained in providing | 24–26,32–35,37,41–43,45,47,48,51–53,58–62,64,72,75,77,79,81, 90,96 | *"This chemist is one of my friends. Hence, I had already decided to consult and buy the medical abortion kit from him. I knew that he, being my friend, would advise me better than other chemists. He would be more reliable and accurate for buying the kit. He could also explain in detail about MA: "he was the ultimate option for me. Therefore, I* |

| Process | Trajectory | Description | ID numbers of studies contributing to the findings | Quotations: First or second order |
|---|---|---|---|---|
| | | safe abortion services including doctors, nurses, midwives, clinicians and nurse-assistants who often provide clandestine abortion services in their clinics and are preferred by women due to low cost, social safety and proximity, traditional herbalists or abortionists who have no medical training but are preferred due to accessibility, social safety and low cost, social networks such as relatives or friends who are trusted to keep the procedure confidential, or self-induced for fear of stigma or in cases of prescribed medical abortion which may be done at home by the woman herself. Abortion seekers may visit multiple providers several times, especially when the abortion is incomplete. | | *planned to buy medical abortion kit from this chemist." (Partner, male, 32 years old, India)*[30]<br><br>*"The doctor is extremely polite and does not discriminate among patients. I like this doctor. I went to her for the first time for my childbirth. She is very impressive and gentle in treating people."(female, unknown age, India)*[41] |
| | | | | |
| **Post abortion care and experience s** | **Post-abortion complications** | Incomplete abortions or abortions with complications were often treated at referral health facilities or hospitals that had the capacity to manage severe complications and provide comprehensive post-abortion care, or in clinics as part of the MA package or as an independent service for post-abortion care. | 6,24,25,30,31,34,36,38,42,43,45,47,53,58–60,72,79–82 | *"I didn't have any intention of visiting the hospital but when I began to bleed, the blood came gradually. About a few weeks later, I began to have severe stomachache, my parents thought it was malaria, so my younger sister went to get a drug for me at the chemist, a white drug sort of, that was what I used, and I was relieved. I couldn't even check the name because of my condition. So, when I used it, I felt ok for about 2 weeks but on Saturday the pains began* |

| Process | Trajectory | Description | ID numbers of studies contributing to the findings | Quotations: First or second order |
|---|---|---|---|---|
| | | | | *again, and I was bleeding that is why my grandma brought me here." (female, 23 years old, Nigeria)*[24]*.*<br><br>*"I came because my bleeding wouldn't stop. It continued for 20 days. I felt weak and was really scared. I told my neighbour about my condition, and she suggested that I consult this doctor. She had consulted her previously. She said the doctor was very good." (female, 23 years old, India)*[30]*.*<br><br>*"I thought that if I don't come to the hospital and get help, I remain in the house I will get worse and die. I would get worse if I didn't tell my family. That's why when I told my family they helped me come to the hospital." (female, 20 years old, Papua New Guinea)*[25]*.* |
| | **Post-abortion contraceptive use** | Only two of the included articles provided information on provision of post-abortion services and counselling on contraception and it was not universally offered to all the women who might have needed the services. | [36,46] | *Not all women received contraceptive counselling after abortion. Some said they were informed about pills, injection, and intrauterine device (IUD), and a few women reported being advised to use traditional methods such as the calendar method (Cambodia)*[46]*.* |

**First order quotes**: study participants quotes; **second order quotes**: author interpretations/explanations; first order quotes are in quotation marks

**Risk of bias assessment**

**NB:** Two papers – Akin 2015 which was a partially randomised controlled trial and Arambepola 2014 which had a quantitative component (unmatched case-control study) had high overall quality (results not shown). Akin 2015 scored 'yes' 7/7 times in JBI assessment for quasi-RCT questions that were applicable to the study design (two questions were not applicable). Arambepola 2014 scored 'yes' 7/8 in the applicable JBI assessment questions for case control studies (two questions were not applicable and it scored unclear for one question).

*ST 4: Quality assessment for the qualitative studies using the CASP appraisal tool for qualitative studies*

| | CASP qualitative assessment questions | | | | | | | | | | |
|---|---|---|---|---|---|---|---|---|---|---|---|
| Study ID | 1 | 2 | 3 | 4 | 5 | 6 | 7 | 8 | 9 | 10 | Overall Quality |
| Sri B et al 2014 | Y | Y | Y | Y | Y | CT | Y | Y | Y | Y | H |
| Chahal et al 2017 | Y | Y | Y | CT | Y | Y | Y | Y | Y | Y | H |
| Gbagbo et al 2020 | Y | Y | Y | Y | Y | CT | Y | Y | Y | Y | H |
| Dahlback et al 2007 | Y | Y | Y | Y | Y | CT | Y | CT | Y | Y | H |
| Dahlback et al 2010 | Y | Y | Y | CT | Y | Y | Y | CT | Y | Y | H |
| Geressu et al 2010 | Y | Y | Y | Y | Y | CT | Y | Y | Y | Y | H |
| Masuda et al 2020 | Y | Y | Y | Y | Y | N | CT | Y | Y | Y | H |
| Lima et al 2020 | Y | Y | CT | N | CT | N | Y | CT | Y | Y | L |
| Marlow et al 2014 | Y | Y | Y | CT | Y | CT | Y | Y | Y | Y | H |
| Oyefara et al 2017 | Y | Y | CT | Y | Y | CT | CT | Y | Y | Y | M |
| Oyeniran et al 2019 | Y | Y | Y | Y | Y | CT | Y | Y | Y | Y | H |
| Rominski et al 2017 | Y | Y | Y | Y | CT | CT | Y | Y | Y | Y | H |
| Vallely et al 2015 | Y | Y | Y | Y | Y | CT | Y | Y | Y | Y | H |
| Solheim et al 2020 | Y | Y | Y | Y | Y | Y | Y | Y | Y | Y | H |
| Tong et al 2012 | Y | Y | Y | N | Y | N | Y | Y | Y | Y | H |
| Coast et al 2016 | Y | Y | Y | Y | Y | CT | Y | Y | Y | Y | H |
| Nourizadeh et al 2020 | Y | Y | Y | Y | Y | Y | CT | Y | Y | Y | H |
| Arambepola et al 2014 | Y | Y | Y | Y | Y | CT | Y | CT | Y | Y | H |
| Arnott et al 2017 | Y | Y | Y | Y | CT | CT | Y | Y | Y | Y | H |
| Bain et al 2019 | Y | Y | Y | Y | Y | CT | Y | Y | Y | Y | H |
| Baum et al 2020 | Y | Y | Y | Y | Y | N | Y | Y | Y | Y | H |
| Berry-Bibee 2018 | Y | Y | Y | Y | Y | CT | Y | CT | Y | Y | H |
| | | | | | | | | | | | |
| Azmat et al 2012 | Y | Y | Y | Y | Y | N | N | CT | Y | Y | M |
| Banerjee et al | Y | Y | Y | CT | CT | N | CT | Y | Y | Y | M |
| Bui et al 2011 | Y | Y | Y | Y | Y | N | CT | Y | Y | Y | M |
| Bury 2012 et al | Y | Y | CT | CT | CT | N | CT | Y | Y | Y | L |
| Chareka et al 2021 | Y | Y | Y | Y | Y | N | Y | Y | Y | Y | H |
| Chiweshe et al 2021 | Y | Y | Y | Y | Y | N | Y | Y | Y | Y | H |

| | CASP qualitative assessment questions | | | | | | | | | | |
|---|---|---|---|---|---|---|---|---|---|---|---|
| Study ID | 1 | 2 | 3 | 4 | 5 | 6 | 7 | 8 | 9 | 10 | Overall Quality |
| Dijk et al 2011 | Y | Y | CT | Y | Y | CT | Y | Y | Y | Y | H |
| Ferrari et al 2020 | Y | Y | Y | Y | Y | N | Y | Y | Y | Y | H |
| Frederico et al 2017 | Y | Y | CT | Y | Y | Y | Y | Y | Y | Y | H |
| Freeman et al 2019 | Y | Y | Y | Y | Y | CT | Y | Y | Y | Y | H |
| Ganatra et al 2002 | Y | Y | Y | Y | Y | N | CT | CT | Y | Y | M |
| Ganatra et al 2010 | Y | Y | Y | Y | Y | N | Y | CT | Y | Y | H |
| Gresh et al 2014 | Y | Y | Y | N | Y | CT | Y | Y | Y | Y | H |
| Harries et al 2021 | Y | Y | Y | Y | Y | N | Y | Y | Y | Y | H |
| Herrera et al 2002 | Y | Y | Y | Y | CT | N | CT | N | CT | Y | L |
| Ituarte et al 2021 | Y | Y | Y | Y | Y | N | Y | CT | Y | Y | H |
| Izugbara et al 2015 | Y | Y | Y | Y | Y | N | Y | CT | Y | Y | H |
| Jejeebhoy et al 2010 | Y | Y | N | CT | N | N | CT | N | N | Y | L |
| Juarez et al 2011 | Y | Y | Y | Y | Y | CT | Y | Y | Y | Y | H |
| Katz et al 2022 | Y | Y | Y | Y | Y | N | Y | Y | Y | Y | H |
| Kebede et al 2018 | Y | Y | Y | Y | Y | Y | Y | Y | Y | Y | H |
| Koster et al 2010 | Y | Y | Y | Y | Y | CT | Y | CT | Y | Y | H |
| Kumi-Kyereme et al 2014 | Y | Y | Y | Y | Y | CT | Y | Y | Y | Y | H |
| Larrea et al 2021 | Y | Y | Y | Y | Y | N | Y | Y | Y | Y | H |
| Loi et al 2018 | Y | Y | Y | Y | Y | Y | Y | Y | Y | Y | H |
| MacFarlane et al 2016 | Y | Y | Y | Y | Y | CT | Y | Y | Y | Y | H |
| Mitchell et al 2010 | Y | Y | Y | Y | Y | N | Y | Y | Y | Y | H |
| Osur et al 2015 | Y | Y | Y | Y | Y | N | CT | CT | Y | Y | M |
| Ouedraogo et al 2020 | Y | Y | Y | Y | Y | Y | Y | Y | Y | Y | H |
| Ouedraogo et al 2020 | Y | Y | Y | Y | Y | Y | Y | Y | Y | Y | H |
| Penfold et al 2018 | Y | Y | N | Y | Y | N | Y | N | Y | Y | M |
| Peres et al 2006 | Y | Y | CT | Y | Y | CT | CT | CT | Y | Y | M |
| Puri et al 2007 | Y | Y | Y | Y | Y | CT | Y | Y | Y | Y | H |
| Ramachandar et al 2004 | Y | CT | N | Y | CT | N | N | N | Y | Y | L |
| Ramos et al 2015 | Y | Y | Y | Y | Y | CT | Y | Y | Y | Y | H |
| Rogers et al 2019 | Y | Y | Y | Y | Y | CT | Y | Y | Y | Y | H |
| Schuster et al 2005 | N | Y | CT | N | CT | Y | N | N | CT | Y | L |
| Srivastava et al 2019 | Y | Y | Y | Y | Y | CT | Y | Y | Y | Y | H |
| Tatum et al 2012 | Y | Y | Y | Y | Y | N | Y | Y | Y | Y | H |

Y= yes; CT = can't tell; N = no; H =high; M = medium; L = low. CASP questionnaire for qualitative studies: 1. Was there a clear statement of the aims of the research? 2. Is a qualitative methodology appropriate? 3. Was the research design appropriate to address the aims of the research? 4. Was the recruitment strategy appropriate to the aims of the research? 5. Was the data collected in a way that addressed the research issue? 6. Has the relationship between researcher and participants been adequately considered? 7. Have ethical issues been taken into consideration? 8. Was the data analysis sufficiently rigorous? 9. Is there a clear statement of findings? 10. How valuable is the research?

*ST 5: Quality assessment for descriptive cross-sectional studies using the JBI tool*

| | JBI assessment questions | | | | | | | | | | |
|---|---|---|---|---|---|---|---|---|---|---|---|
| Study ID | 1 | 2 | 3 | 4 | 5 | 6 | 7 | 8 | 9 | Overall appraisal: | Overall quality |
| Abdella et al 2011 | Y | Y | Y | Y | Y | U | Y | Y | Y | Include | H |
| Berry-Bibee et al 2018 | Y | Y | U | Y | Y | N | Y | Y | Y | Include | H |
| Bui K et al 2010 | Y | Y | U | Y | Y | U | U | Y | N | Include | M |
| Calves et al 2002 | Y | Y | U | Y | Y | N | Y | Y | Y | Include | H |
| Coast et al 2016 | U | N | U | Y | Y | Y | Y | Y | NA | Include | M |
| Chunuan et al 2012 | Y | Y | Y | Y | Y | Y | U | Y | Y | Include | H |
| Dahlback et al 2007 | Y | Y | U | Y | U | Y | Y | Y | NA | Include | H |
| Dahlback et al 2010 | Y | Y | U | Y | Y | Y | U | Y | N | Include | M |
| Dhillon et al 2004 | Y | Y | Y | Y | U | N | Y | Y | U | Include | M |
| Ekanem et al 2007 | U | U | U | Y | Y | Y | Y | Y | U | Include | M |
| Ganatra et al 2002 | Y | Y | U | Y | Y | N | Y | Y | Y | Include | H |
| Gbagbo et al 2020 | Y | Y | Y | Y | Y | Y | Y | Y | Y | Include | H |
| Korejo et al 2003 | Y | N | NA | Y | Y | N | U | Y | NA | Include | M |
| Pilecco et al 2015 | Y | Y | Y | Y | U | Y | U | Y | Y | Include | H |
| Rachana et al 2007 | U | U | U | U | U | Y | Y | Y | U | Include | L |
| Thapa et al 2013 | U | N | Y | N | Y | N | N | Y | Y | Include | L |

Y = yes; U = unknown; NA = not applicable; N = no; H = high; L = low; 1. Was the sample frame appropriate to address the target population? 2. Were study participants sampled in an appropriate way? 3. Was the sample size adequate? 4. Were the study subjects and the setting described in detail? 5. Was the data analysis conducted with sufficient coverage of the identified sample? 6. Were valid methods used for the identification of the condition? 7. Was the condition measured in a standard, reliable way for all participants? 8. Was there appropriate statistical analysis? 9. Was the response rate adequate, and if not, was the low response rate managed appropriately?

*ST 6: Quality assessment for analytical cross-sectional studies using the JBI tool*

| | JBI assessment questions | | | | | | | | | |
|---|---|---|---|---|---|---|---|---|---|---|
| Study ID | 1 | 2 | 3 | 4 | 5 | 6 | 7 | 8 | Overall appraisal: | Overall quality |
| Biney et al 2016 | Y | Y | N | Y | Y | Y | Y | Y | Include | H |
| Bury et al 2012 | U | U | NA | N | NA | NA | U | Y | Include | L |
| Byrne et al 2021 | Y | Y | Y | Y | Y | Y | Y | Y | Include | H |
| Jejeebhoy et al 2010 | Y | Y | U | Y | U | U | U | U | Include | L |
| Klu et al 2020 | Y | Y | Y | Y | Y | Y | Y | Y | Include | H |
| Kumi-Kyereme et al 2014 | Y | Y | Y | Y | Y | Y | U | Y | Include | H |
| Mitchell et al 2010 | Y | Y | Y | Y | Y | Y | Y | Y | Include | H |
| Osur et al 2015 | Y | U | NA | Y | Y | Y | Y | Y | Include | H |
| Puri et al 2007 | Y | N | Y | Y | NA | Y | N | Y | Include | M |

| | JBI assessment questions | | | | | | | | | |
|---|---|---|---|---|---|---|---|---|---|---|
| Study ID | 1 | 2 | 3 | 4 | 5 | 6 | 7 | 8 | Overall appraisal: | Overall quality |
| Sharma et al 2019 | Y | Y | N | Y | Y | Y | U | Y | Include | H |
| Tamang et al 2012 | Y | Y | Y | U | Y | Y | Y | Y | Include | H |
| Zavier et al 2019 | Y | Y | Y | Y | NA | NA | Y | Y | Include | H |
| Zuo et al 2012 | Y | Y | NA | Y | Y | Y | U | Y | Include | H |

Y = yes, U = unknown; NA = not applicable; N = no; H = high; L = low; 1. Were the criteria for inclusion in the sample clearly defined? 2. Were the study subjects and the setting described in detail? 3. Was the exposure measured in a valid and reliable way?

4. Were objective, standard criteria used for measurement of the condition? 5. Were confounding factors identified? 6. Were strategies to deal with confounding factors stated? 7. Were the outcomes measured in a valid and reliable way? 8. Was appropriate statistical analysis used?

*ST 7: Abortion decision-making trajectories*

| Process | Trajectory | Description | ID numbers of studies contributing to the findings | Quotations: First or second order |
|---|---|---|---|---|
| Pregnancy awareness | **Pregnancy awareness** | The abortion decision-making trajectory starts with the discovery of pregnancy which may be expected or unexpected. Unexpected pregnancies are likely preceded by "unpreparedness" which may include casual sex or non-use of modern contraceptives. For some women, the pregnancy may be discovered through a pregnancy test or after missing periods. The pregnancy may have been planned by one or both partners. In other cases, the pregnancy may not have been planned at all. | [1–12] | *"I found out that I have a son. I had a casual relationship...with a woman I knew...that any time she or I felt like it [having sex], one person would say, "hey, should we see each other" ... and the other person would say, "yes, okay" ... One day she showed up and said, "I'm late. I took a test and I think I'm pregnant." So we went to the doctor, they confirmed it...she was one and a half months along...The doctor talked to us and said everything, "The decision is for both of you to make. If you want to make the decision to not have it, you have 2 - 3 more weeks...You can come to my office or to another office, for them to do an aspiration and in 3 - 4 hours, you'll walk out of my office without a problem ... so talk about it and make the best decision...We talked and we agreed that she wasn't going to have it ... But then she had it. (Male, 32 years old, Mexico)*[6]*.*<br><br>*"When I opened the test and saw that it was positive, I called him, he came at the same time. I was upset, he told me not to worry that everything was going to be fine, that he doesn't earn much money, but what he earns will allow us to manage the pregnancy [...]. When I left him, I went straight to my friends to say that it is positive, that my boyfriend wants me to keep but I want to have an abortion. She agreed that I should have an abortion and she already* |

| Process | Trajectory | Description | ID numbers of studies contributing to the findings | Quotations: First or second order |
|---|---|---|---|---|
| | | | | *knew a good method to have a safe abortion [...] When I explained to him [her boyfriend] that I was going to have an abortion, he didn't want it. [...] But then, as we were talking, he calmed down a bit, he changed his mind."(Female, 23 years old, Burkina Faso)*[1]. |
| | **Self-reflection** | While some women may be stress-free during this phase, women may alternate between stressful and stress-free periods. The reaction to a pregnancy may be that of shock, surprise, and stress or ambivalent with periods of stress alternating with stress-free situations. The phase is variable and can take a few hours to weeks or even months. | 1,11–17 | *"I was still deciding what to do; I was still doubting. So many things run into my mind until I come with the decision to do [---] to [---] to [---] end the pregnancy. At that time [---] [I] even think I lost [---][weight] cause [because of the] stress I have [---]having so many stress [---] losing weight cause of the stress."(Female, 22 years old, Kenya)*[14]. <br><br> *Women shared that this time of reflection enables them to decide what they want before the personal decision becomes subject to community perspectives (both supportive and in opposition to the decision(Kenya)*[14]. |
| | **Abortion contemplation /initial decision-making** | Following the phase of self-reflection, women may make an initial abortion decision, a decision most often influenced by the environment in which she lives and the perceived reactions of her social networks to the pregnancy. The initial decision may be pregnancy termination, pregnancy continuation or ambivalence | 1,2,6–9,11,14,15,17–20 | *"At first, I decided I wanted to continue... so a few weeks went by and I talked to my grandmother about it.... She helped me and told me to [have the baby]. But when I talked to my mom, things changed. She told me I would lose lots of things, that I would lose the house, school, that they wouldn't give me anything. Then things started to look pretty bleak.... That same night, I got my things together* |

| Process | Trajectory | Description | ID numbers of studies contributing to the findings | Quotations: First or second order |
|---|---|---|---|---|
| | | about the pregnancy but women may go through all these phases iteratively at different time intervals. While the perceived reaction of the social network to the pregnancy may influence the initial abortion decision, adult women often make their decisions independently. Adolescents may defer the initial decision to their parents often due to fear of their parents' reaction to the pregnancy or because they are resigned to the fact that the decision will be made by parents irrespective of their choice. However, even in cases where adolescents may make the initial decision on abortion, parents often override this initial decision if it is mostly at variance with their choice. | | *and said to my dad, "You know what? I'm going to live with [my boyfriend]," but my dad got mad and started to yell. "No, no, no. I don't want you to have [the baby]." [female, 17-year-old, Mexico]*[9]. <br><br> *"At first, I didn't dare talk about it with anyone, even my boyfriend, I didn't want to tell him because I was thinking he would abandon me. I've been thinking for several days, how am I going to do that [tell him]? Finally, I decided that I had to talk to my boyfriend about it since he is also affected (female, 20 years old, Burkina Faso )*[1]. |
| | **Disclosure and seeking support** | Following the initial abortion decision is the phase of disclosure and seeking support. The pregnant woman may disclose to or conceal the pregnancy and the initial decision from her social networks including partner, parents, siblings, teachers, and other close friends. This disclosure and seeking support depend on the level of trust and expected support from the persons to whom the pregnancy or its decision is disclosed. | 1,7,9,11,12,14,16,17,21–24 | *"I was doubting what to do and on the other hand afraid of sharing with anybody. I believed if I share it with so many people some people will give me other advice, some will give me this; that's why I ended up sharing with my sister that I'm staying with because I trusted her" (female, 22 years old, Kenya)*[14]. <br><br> *"I did not ask someone for advice because if you ask one they will start talking about it and everybody will know* |

| Process | Trajectory | Description | ID numbers of studies contributing to the findings | Quotations: First or second order |
|---|---|---|---|---|
| | | When the level of trust and support is perceived to be low or non-existent, women may not disclose the pregnancy to their social networks. Some women may only disclose to a person who is expected to make the decision or provide emotional support for the abortion process. For example, adolescents may disclose their pregnancies to their parents who they expect will make decisions on their behalf but may also conceal the pregnancy due to fear or perceived opposition to their initial decision. Disclosure or concealment among adult married women is dependent on level of support from their partner and relationship stability. | | *about it, so I was afraid to talk about it to someone [---] and maybe it will go back to my partner, and I didn't want that to happen." (female, 32 years old, Kenya)*[14]. |
| | **Negotiations/ bargaining** | The woman and the actors to whom the abortion decision has been disclosed to then enter a stage of negotiation in cases of discordance and indecision where the woman tries to convince her social networks to agree to her initial decision. | 1,2,7,9,14,25,26 | *"She is the one who at first told me it was not the baby's fault and that it was me who had been irresponsible.... My friend was basically against the idea of my getting an abortion.... She said, "And what if you do not make it through? What will I do without you?" I said, "Nothing is going to happen. I am going to be fine." [female, 16-year-old, Mexico]*[9]. |
| | **Final decision** | With the completion of the negotiations, the decision-making process may enter | 1–3,5,6,8–12,14–20,22–51 | *"Then I spoke to my boyfriend and he said, "I am going to do the right thing. We are going to talk to your father." Then* |

| Process | Trajectory | Description | ID numbers of studies contributing to the findings | Quotations: First or second order |
|---|---|---|---|---|
| | | the second stage which begins with the final abortion decision – whether to keep or terminate the pregnancy. The decision may be made jointly with partner/parents, by the woman alone – sometimes without even informing the partner or parents, by the partner alone or primarily, or by others such as parents, in-laws, relatives, friends, and teachers. Even in cases in which the majority of the decisions were made by women, men or parents still influenced their decision. Thus, decisions made by women were either passive in which the woman agreed to the decision imposed on her by others or active in which she actively participated in the decision-making processes. In cases where women were excluded or played a very limited role, threats, coercion, violence, or trickery were often employed by partners or parents to get the woman to terminate the pregnancy reflecting a lack control and power of women in making decisions regarding their own pregnancy. | | *[my father] sent for my boyfriend and asked, "How is it going to be? What are you going to do?" My boyfriend told him, "I am going to be responsible. I am going to do what I have to do." [female, 17-year-old, Mexico]*[9].<br><br>*"When I had my first pregnancy, I didn't tell my husband about it, so I went to abort it…I thought he was going to get angry because he already told me that he is not ready for pregnancy" [female, 22 years old, Nigeria]*[2].<br><br>*"I wanted to keep it but he said, "Why are you so eager to keep it? It must be someone else's. If it is mine, you will do as I say. After that what could I do?" (female, 19-year-old, India)*[15].<br><br>*"It was great, he said, "stop it, what you decided, it's decided. If you want to have it, then you will have it. If you want to have an abortion, I'm with you, don't worry." Still, he is so friendly and supportive of me." [female, 23 years old, Brazil]*[29].<br><br>*"My dad told me he would disown me if I don't get an abortion" [female, 17 years old, Ghana]*[27]. |

| Process | Trajectory | Description | ID numbers of studies contributing to the findings | Quotations: First or second order |
|---|---|---|---|---|
| | **Access and information** | Once the final decision has been made, women and their social networks such as parents or partners consult multiple sources to obtain information about availability, affordability, safety or accessibility to abortion services. Various sources of information include skilled abortion providers, chemists or unskilled abortion providers, intermediaries or brokers, partners, social networks such as friends, parents, teachers, and relatives, the media and internet or school. | 2–5,8,10–12,14,16,23,24,29,30,33,34,37,41,43,48,51–53 | *“Once you get pregnant you will know all of those places and the ones who are selling those drugs. You will have their contacts as well as doctor's contacts. You can even find students who are agents for these doctors, and they will direct you to them as they know who are good at this job.” (female, 21 years, Tanzania)*[4]. <br><br> *"My partner began investigating options on the Internet. He found the government webpage and called the number. They told him where the clinics were, what the requirements were, and that you don't necessarily have to live here in Mexico City, and that in fact a lot of people come from other states."[female, 21 years old, Mexico]*[53]. <br><br> *"I knew that I couldn't ask for information about abortion services from people who I am close to and yet keep my pregnancy secret. [...] I did not know even how to raise the issue. But the delala (abortion intermediaries) was there. He approached me while sitting outside the hospital compound. He himself brought up the issue and gave me the information about abortion service providers. Back then I didn't know that he was a delala. He was nice. He promised to find me a provider who gives clean services for a fair price."(female, 19 years old, Ethiopia)*[52]. |

| Process | Trajectory | Description | ID numbers of studies contributing to the findings | Quotations: First or second order |
|---|---|---|---|---|
| | **Methods** | Methods used to terminate the pregnancy are either safe or unsafe. Safe methods used include medical abortion (MA) which is preferred by women for its ease and convenience of use, avoidance of painful surgical procedures and because of the control the woman has over the process. Surgical methods including both manual vacuum aspiration (MVA) and dilatation and curettage (D&C) are preferred by some women considering them to be a one-time effective method for terminating the pregnancy with no requirement for visiting the hospital again. The unsafe methods include traditional methods that involve ingestion of over-the-counter drugs such as chloroquine, quinine, and antibiotics, local herbs and roots, insertion of sticks and cassava roots to the cervix by unskilled women or applying blunt manual force to the abdomen. | 2–5,7,8,10–12,14,15,17,19,21–24,27–30,33,35–44,46,50,51,54–61 | *"Yes. We prefer the [MA] (medical abortion) kit only; it is more trustworthy and we would visit the same store for purchasing the kit in future."(male partner, 31 years, India)*[8]*.*<br><br>*"She asked me to wait and said that the abortion would definitely occur. But my family members started shouting and saying nothing is happening with the tablet and the days (gestational age) are increasing, so I decided to get admitted for a surgical abortion" (female, 35 years old, India)*[37]*.*<br><br>*Unskilled old women who insert sticks or roots of cassava into the cervix carry out another type of traditional method used to provoke unsafe abortion. When left in place, the cassava dilates the cervix and stimulates uterine contractions. Two girls who aborted at home were admitted to the hospital with severe infections after having cassava sticks or roots inserted into their cervices (Zambia)*[55]*.* |
| | **Place** | Abortion services are obtained at various places including government/public facilities which are considered to be generally safe, cost-effective and have a good reputation. These could be referral centres for management of post abortion | 2,5,10–12,17,19,21–23,25,28–30,33,36,40–43,46,50,51,54,55,60–63 | *"I never knew one could stay in the comfort of your house. I think I really never knew about all that before, all my mind was that abortion must be done in the Health Care for Women International hospital or you start to use all those local concoctions that you don't even know what the future* |

| Process | Trajectory | Description | ID numbers of studies contributing to the findings | Quotations: First or second order |
|---|---|---|---|---|
| | | complications arising from clandestine and unsafe abortion, private licensed clinics and health facilities which may be run by NGOs and offer subsidised, safe and legal abortion services, private unlicensed clinics that often offer illegal, unsafe, clandestine but low cost abortion services, homes of traditional abortion providers which are preferred for cost-effectiveness, social safety, and ease of access and availability, or at own home, school or neighbour's home which are considered comfortable and permitted for convenient use of medical abortion. Government facilities and private licensed clinics and health facilities often provide safe and legal abortions while all the other abortion services mostly provide unsafe, clandestine and often illegal abortion services. | | *holds." (female, 29 years old, Nigeria)*[5]. <br><br> *"The operating theatre is very big, equipped with powerful lighting, and the table is very big and the sheets are very clean. There was an anaesthetist and I could see so much fancy medical equipment inside the theatre. The doctor was very polite and the theatre staff are equally good and kind to the patients. Doctor "kku rasianna kai" (The doctor has lucky hands). I was asked to change my clothes and they provided me with a neatly pressed hospital gown that I had to wear before I got on the table. The theatre surgeon was very polite and kind. Although I got restless waiting for a long time, still I was pleased with the overall treatment that was given to me. I did not have any complications after getting abortion and sterilisation.(female, unknown age, India)"*[21]. |
| | **Providers** | The providers of abortion services include skilled and trained health workers who provided safe abortion services and are often obstetricians/gynaecologists, other medical doctors, nurses or midwives trained in provision of abortion services, health workers not trained in providing safe abortion services including doctors, | 2–4,10–12,15,17,21–23,28–30,33–35,40–44,46,51,55,57,59,61,63,64 | *"This chemist is one of my friends. Hence, I had already decided to consult and buy the medical abortion kit from him. I knew that he, being my friend, would advise me better than other chemists. He would be more reliable and accurate for buying the kit. He could also explain in detail about MA: "he was the ultimate option for me. Therefore, I planned to buy medical abortion kit from this chemist."* |

| Process | Trajectory | Description | ID numbers of studies contributing to the findings | Quotations: First or second order |
|---|---|---|---|---|
| | | nurses, midwives, clinicians and nurse-assistants who often provide clandestine abortion services in their clinics and are preferred by women due to low cost, social safety and proximity, traditional herbalists or abortionists who have no medical training but are preferred due to accessibility, social safety and low cost, social networks such as relatives or friends who are trusted to keep the procedure confidential, or self-induced for fear of stigma or in cases of prescribed medical abortion which may be done at home by the woman herself. Abortion seekers may visit multiple providers several times, especially when the abortion is incomplete. | | *(Partner, male, 32 years old, India)*[8]<br><br>*"The doctor is extremely polite and does not discriminate among patients. I like this doctor. I went to her for the first time for my childbirth. She is very impressive and gentle in treating people."(female, unknown age, India)*[21] |
| | | | | |
| **Post abortion care and experience s** | **Post-abortion complications** | Incomplete abortions or abortions with complications were often treated at referral health facilities or hospitals that had the capacity to manage severe complications and provide comprehensive post-abortion care, or in clinics as part of the MA package or as an independent service for post-abortion care. | 2,3,8,9,12,16,18,22,23,28,29,35,40–42,51,54,59,60,63,65 | *"I didn't have any intention of visiting the hospital but when I began to bleed, the blood came gradually. About a few weeks later, I began to have severe stomachache, my parents thought it was malaria, so my younger sister went to get a drug for me at the chemist, a white drug sort of, that was what I used, and I was relieved. I couldn't even check the name because of my condition. So, when I used it, I felt ok for about 2 weeks but on Saturday the pains began* |

| Process | Trajectory | Description | ID numbers of studies contributing to the findings | Quotations: First or second order |
|---|---|---|---|---|
| | | | | *again, and I was bleeding that is why my grandma brought me here." (female, 23 years old, Nigeria)*[2].<br><br>*"I came because my bleeding wouldn't stop. It continued for 20 days. I felt weak and was really scared. I told my neighbour about my condition, and she suggested that I consult this doctor. She had consulted her previously. She said the doctor was very good." (female, 23 years old, India)*[8].<br><br>*"I thought that if I don't come to the hospital and get help, I remain in the house I will get worse and die. I would get worse if I didn't tell my family. That's why when I told my family they helped me come to the hospital." (female, 20 years old, Papua New Guinea)*[3]. |
| | **Post-abortion contraceptive use** | Only two of the included articles provided information on provision of post-abortion services and counselling on contraception and it was not universally offered to all the women who might have needed the services. | [16,24] | *Not all women received contraceptive counselling after abortion. Some said they were informed about pills, injection, and intrauterine device (IUD), and a few women reported being advised to use traditional methods such as the calendar method (Cambodia)*[24]. |

**First order quotes**: study participants quotes; **second order quotes**: author interpretations/explanations; first order quotes are in quotation marks

## References


1. Ouedraogo R, Senderowicz L, Ngbichi C. “I wasn’t ready”: abortion decision-making pathways in Ouagadougou, Burkina Faso. Int J Public Health. 2020;65(4 PG-477–486):477–86.

2. Oyeniran AA, Bello FA, Oluborode B, Awowole I, Loto OM, Irinyenikan TA, et al. Narratives of women presenting with abortion complications in Southwestern Nigeria: A qualitative study. PLoS One. 2019;14(5 PG-e0217616):e0217616.

3. Vallely LM, Homiehombo P, Kelly-Hanku A, Whittaker A. Unsafe abortion requiring hospital admission in the Eastern Highlands of Papua New Guinea - A descriptive study of women’s and health care workers’ experiences. Reprod Health. 2015;12(1 PG-).

4. Solheim IH, Moland KM, Kahabuka C, Pembe AB, Blystad A. Beyond the law: Misoprostol and medical abortion in Dar es Salaam, Tanzania. Soc Sci Med. 2020;245(PG-):112676.

5. Baum SE, Ramirez AM, Larrea S, Filippa S, Egwuatu I, Wydrzynska J, et al. “It’s not a seven-headed beast”: abortion experience among women that received support from helplines for medication abortion in restrictive settings. Health Care Women Int. 2020;(PG-).

6. Juarez F, Bayer AM. “Without a plan” but “keeping on track”: views on contraception, pregnancy and abortion in Mexico City. Glob Public Health. 2011;6 Suppl 1(PG-S90-110):S90-110.

7. Peres SO, Heilborn ML. Cogitação e prática do aborto entre jovens em contexto de interdição legal: O avesso da gravidez na adolescência. Cad Saude Publica. 2006;22(7):1411–20.

8. Srivastava A, Saxena M, Percher J, Diamond-Smith N. Pathways to seeking medication abortion care: A qualitative research in Uttar Pradesh, India. PLoS One. 2019;14(5):e0216738.

9. Tatum C, Rueda M, Bain J, Clyde J, Carino G. Decision-making regarding unwanted pregnancy among adolescents in Mexico City: A qualitative study. Stud Fam Plann. 2012;43(1 PG-43–56):43–56.

10. Ituarte ML, López-Gómez A. Adolescents faced with the decision to terminate a pregnancy in a context of legal abortion. Cad Saude Publica. 2021;37(2):e00235219.

11. Larrea S, Hidalgo C, Jacques-Aviñó C, Borrell C, Palència L. “No one should be alone in living this process”: trajectories, experiences and user’s perceptions about quality of abortion care in a telehealth service in Chile. Sex Reprod Heal Matters. 2022;29(3).

12. Ferrari W, Peres S. Itinerários de solidão: aborto clandestino de adolescentes de uma favela da Zona Sul do Rio de Janeiro, Brasil. Cad Saude Publica. 2020;36Suppl 1(Suppl 1):e00198318.

13. Puri M, Ingham R, Matthews Z. Factors Affecting Abortion Decisions among Young Couples in Nepal. J Adolesc Heal. 2007 Jun;40(6 PG-535–542):535–42.

14. Rehnström Loi U, Lindgren M, Faxelid E, Oguttu M, Klingberg-Allvin M. Decision-making preceding induced abortion: A qualitative study of women’s experiences in Kisumu, Kenya 11 Medical and Health Sciences 1117 Public Health and Health Services. Reprod Health. 2018;15(1):1–12.

15. Bela G, Siddhi H, Ganatra B, Hirve S, Bela G, Siddhi H. Induced abortions among adolescent women in rural Maharashtra, India. Spec issue Abort women decide. 2002;10(19 PG-76–85):76–85.

16. Penfold S, Wendot S, Nafula I, Footman K. A qualitative study of safe abortion and post-abortion family planning service experiences of women attending private facilities in Kenya.

Reprod Health. 2018;15(1 PG-):70.

17. Chi BK, Rasch V, Thi Thuy Hanh N, Gammeltoft T. Pregnancy decision-making among HIV positive women in Northern Vietnam: reconsidering reproductive choice. Anthropol Med. 2011;18(3):315–26.

18. Sri B. S, Ravindran TKSS, B SS, Ravindran TKSS, Sri SB, Ravindran TKSS, et al. Safe, accessible medical abortion in a rural Tamil Nadu clinic, India, but what about sexual and reproductive rights? Reprod Health Matters. 2015;22(PG-134-143):134–43.

19. Frederico M, Michielsen K, Arnaldo C, Decat P. Factors influencing abortion decision-making processes among young women. Int J Environ Res Public Health. 2018;15(2 PG-).

20. Kumi-Kyereme A, Gbagbo FY, Amo-Adjei J. Role-players in abortion decision-making in the Accra Metropolis, Ghana. Reprod Health. 2014;11(1 PG-):70.

21. Ramachandar L, Pelto PJ. Abortion providers and safety of abortion: A community-based study in a rural district of Tamil Nadu, India. Reprod Health Matters. 2004;12(24 SUPPL. PG-138–146):138–46.

22. Chiweshe MT, Fetters T, Coast E. Whose bodies are they? Conceptualising reproductive violence against adolescents in Ethiopia, Malawi and Zambia. Agenda. 2021;35(3):12–23.

23. Heilborn ML, Cabral C da S, Brandão ER, Faro L, Cordeiro F, Azize RL. Itinerários abortivos em contextos de clandestinidade na cidade do Rio de Janeiro - Brasil. Cienc e Saude Coletiva. 2012;17(7):1699–708.

24. Masuda C, Oreglia E, Ly S, McLaren M, Free C, Tijamo C, et al. Family planning practices of women working in the Cambodian garment industry: A qualitative study. Contracept Reprod Med. 2020;5(1):11.

25. Freeman E, Coast E, Murray SF, Freeman BE, Murray SF. Men's roles in women's abortion trajectories in Urban Zambia. Int Perspect Sex Reprod Health. 2017 Jun;43(2):89–98.

26. Osur J, Orago A, Mwanzo I, Bukusi E. Social networks and decision making for clandestine unsafe abortions: Evidence from Kenya. Afr J Reprod Health. 2015 Mar;19(1 PG-34–43):34–43.

27. Engelbert Bain L, Zweekhorst MBMM, Amoakoh-Coleman M, Muftugil-Yalcin S, Omolade AI-OAI-OAI-OO, Becquet R, et al. To keep or not to keep? Decision making in adolescent pregnancies in Jamestown, Ghana. PLoS One. 2019;14(9):1–18.

28. Dahlbäck E, Maimbolwa M, Yamba CB, Kasonka L, Bergström S, Ransjö-Arvidson A-B. Pregnancy loss: spontaneous and induced abortions among young women in Lusaka, Zambia. Cult Health Sex. 2010;12(3 PG-247–262):247–62.

29. Lima NDF, Cordeiro R de LM. "A minha vida não pode parar": itinerários abortivos de mulheres jovens. Rev Estud Fem. 2020;28(1):1–13.

30. Marlow HM, Wamugi S, Yegon E, Fetters T, Wanaswa L, Msipa-Ndebele S. Women's perceptions about abortion in their communities: Perspectives from western Kenya. Reprod Health Matters. 2014 May;22(43):149–58.

31. Tong WT, Low WY, Wong YL, Choong SP, Jegasothy R. Exploring pregnancy termination experiences and needs among Malaysian women: A qualitative study. BMC Public Health. 2012;12(1 PG-).

32. Nourizadeh R, Mohammadi I, Ahmad S, Baghestani R. Explaining the decision-making process

for abortion or persistence in women with unwanted pregnancies : a basic theory study. 2020;25(4).

33. Arambepola C, Rajapaksa LC. Decision making on unsafe abortions in Sri Lanka: a case-control study. Reprod Health. 2014;11(1 PG-):91.

34. Arnott G, Tho E, Guroong N, Foster AM. To be, or not to be, referred: A qualitative study of women from Burma's access to legal abortion care in Thailand. PLoS One. 2017;12(6 PG-):e0179365.

35. Berry-Bibee EN, St Jean CJ, Nickerson NM, Haddad LB, Alcime MM, Lathrop EH. Self-managed abortion in urban Haiti: A mixed-methods study. BMJ Sex Reprod Heal. 2018;44(3 PG-193–199):193–9.

36. Bury L, Aliaga Bruch S, MacHicao Barbery X, Garcia Pimentel F. Hidden realities: What women do when they want to terminate an unwanted pregnancy in Bolivia. Int J Gynecol Obstet. 2012;118(PG-S4-9):S4-9.

37. Ganatra B, Kalyanwala S, Elul B, Coyaji K, Tewari S. Understanding women's experiences with medical abortion: In-depth interviews with women in two Indian clinics. Glob Public Health. 2010;5(4 PG-335–347):335–47.

38. Gresh A, Maharaj P. Termination of pregnancy: perspectives of female students in Durban, South Africa. African Popul Stud / Etude la Popul Africaine. 2014;28(Supp.1 PG-681-690):681–90.

39. Jejeebhoy SJ, Kalyanwala S, Zavier AFJFF, Kumar R, Jha N. Experience seeking abortion among unmarried young women in Bihar and Jharkhand, India: Delays and disadvantages. Reprod Health Matters. 2010;18(35):163–74.

40. Schuster S. Abortion in the moral world of the Cameroon Grassfields. Reprod Health Matters. 2005;13(26 PG-130–138):130–8.

41. Chareka S, Crankshaw TL, Zambezi P, S. C, T.L. C, Zambezi P. AO - Crankshaw TL. O https://orcid. org/000.-0001-6974-1593. Economic and social dimensions influencing safety of induced abortions amongst young women who sell sex in Zimbabwe. Sex Reprod Heal Matters. 2021;29(1):1881209.

42. Harries J, Daskilewicz K, Bessenaar T, Gerdts C, J. H, K. D, et al. Understanding abortion seeking care outside of formal health care settings in Cape Town, South Africa: a qualitative study. Reprod Health. 2021;18(1):1–8.

43. Katz AJ, Ramirez AM, Bercu C, Filippa S, Dirisu O, Egwuatu I, et al. "I just have to hope that this abortion should go well": Perceptions, fears, and experiences of abortion clients in Nigeria. PLoS One. 2022;17(2 February):1–16.

44. Anne-Emmanuele C, Calves A-E, Calvès A-E, Anne-Emmanuele C. Abortion risk and decisionmaking among young people in urban Cameroon. Stud Fam Plann. 2002 Sep;33(3):249–60.

45. Thapa S, Neupane S. Abortion clients of a public-sector clinic and a non-governmental organization clinic in Nepal. J Health Popul Nutr. 2013;31(3 PG-376):376.

46. Chunuan S, Kosunvanna S, Sripotchanart W, Lawantra J, Lawantrakul J, Pattrapakdikul U, et al. Characteristics of Abortions in Southern Thailand. Pacific Rim Int J Nurs Res. 2012;16(2 PG-97–112):97–112.

47. Banerjee SK, Andersen KL, Baird TL, Ganatra B, Batra S, Warvadekar J. Evaluation of a multi-

pronged intervention to improve access to safe abortion care in two districts in Jharkhand. BMC Health Serv Res. 2014;14(1 PG-227):227.

48. Zuo X, Yu C, Lou C, Tu X, Lian Q, Wang Z. Factors affecting delay in obtaining an abortion among unmarried young women in three cities in China. Asia-Pacific Popul J. 2015;30(1 PG-35–50):35–50.

49. Bùi KC, Gammeltoft T, Nguyen TTN, Rasch V, Bui KC, Gammeltoft T, et al. Induced abortion among HIV-positive women in Quang Ninh and Hai Phong, Vietnam. Trop Med Int Health. 2010;15(10):1172–8.

50. Korejo R, Noorani KJ, Bhutta S. Sociocultural determinants of induced abortion. J Coll Physicians Surg Pakistan. 2003;13(5):260–2.

51. Azmat SK, Shaikh BT, Mustafa G, Hameed W, Bilgrami M. Delivering post-abortion care through a community-based reproductive health volunteer programme in Pakistan. J Biosoc Sci. 2012 Nov;44(6):719–31.

52. Kebede MT, Middelthon A-LL, Hilden PK. Negotiating the social and medical dangers of abortion in Addis Ababa: An exploration of young, unmarried women's abortion-seeking journeys. Health Care Women Int. 2018;39(2):186–207.

53. Dijk MG van, Arellano Mendoza LJ, Arangure Peraza AG, Toriz Prado AA, Krumholz A, Yam EA, et al. Women's experiences with legal abortion in Mexico City: a qualitative study. Stud Fam Plann. 2011;42(3 PG-167–174):167–74.

54. Coast E, Murray SF. "These things are dangerous": Understanding induced abortion trajectories in urban Zambia. Soc Sci Med. 2016;153(PG-201-209):201–9.

55. Dahlbäck E, Maimbolwa M, Kasonka L, Bergström S, Ransjö-Arvidson AB. Unsafe induced abortions among adolescent girls in Lusaka. Health Care Women Int. 2007;28(7 PG-654–676):654–76.

56. Geressu T, Gebrehiwot Y, Edelman A, Abdella A, Alemayehu T, Geressu T, et al. Second-Trimester Abortion: Current practices and barriers to service in Ethiopia. Ipas. 2010;(PG-).

57. Izugbara CO, Egesa C, Okelo R. "High profile health facilities can add to your trouble": Women, stigma and un/safe abortion in Kenya. Soc Sci Med. 2015;141(PG-9-18):9–18.

58. Koster W. Linking two opposites of pregnancy loss: Induced abortion and infertility in Yoruba society, Nigeria. Soc Sci Med. 2010;71(10):1788–95.

59. Mitchell EMHH, Kwizera A, Usta M, Gebreselassie H. Choosing early pregnancy termination methods in Urban Mozambique. Soc Sci Med. 2010;71(1 PG-62–70):62–70.

60. Ramos S, Romero M, Aizenberg L. Women's experiences with the use of medical abortion in a legally restricted context: The case of Argentina. Reprod Health Matters. 2015;22(44 PG-4–15):4–15.

61. Byrne ME, Omoluabi E, OlaOlorun FM, Moreau C, Bell SO. Determinants of women's preferred and actual abortion provision locations in Nigeria. Reprod Health. 2021;18(1):1–9.

62. Dhillon BS, Chandhiok N, Kambo I, Saxena NC. Induced abortion and concurrent adoption of contraception in the rural areas of India (an ICMR task force study). Indian J Med Sci. 2004;58(11 PG-478–484):478–84.

63. Chahal H, Mumtaz Z. Abortion and fertility control in Pakistan: The role of misoprostol. J Fam Plan Reprod Heal Care. 2017;43(4 PG-274–280):274–80.

64. Ekanem EI, Etuk SJ, Ekabua JE, Iklaki C. Clinical presentation and complications in patients with unsafe abortions in University of Calabar Teaching Hospital, Calabar, Nigeria. Niger J Med J Natl Assoc Resid Dr Niger. 2009;18(4):370–4.

65. Rominski SD, Lori JR, Morhe ESK. “My friend who bought it for me, she has had an abortion before.” The influence of Ghanaian women’s social networks in determining the pathway to induced abortion. J Fam Plan Reprod Heal Care. 2017;43(3 PG-216–221):216–21.

*ST 8: Subgroup analysis by a country's income status of abortion decision-making involvement by the woman alone, overall woman’s involvement and overall male partner’s involvement*

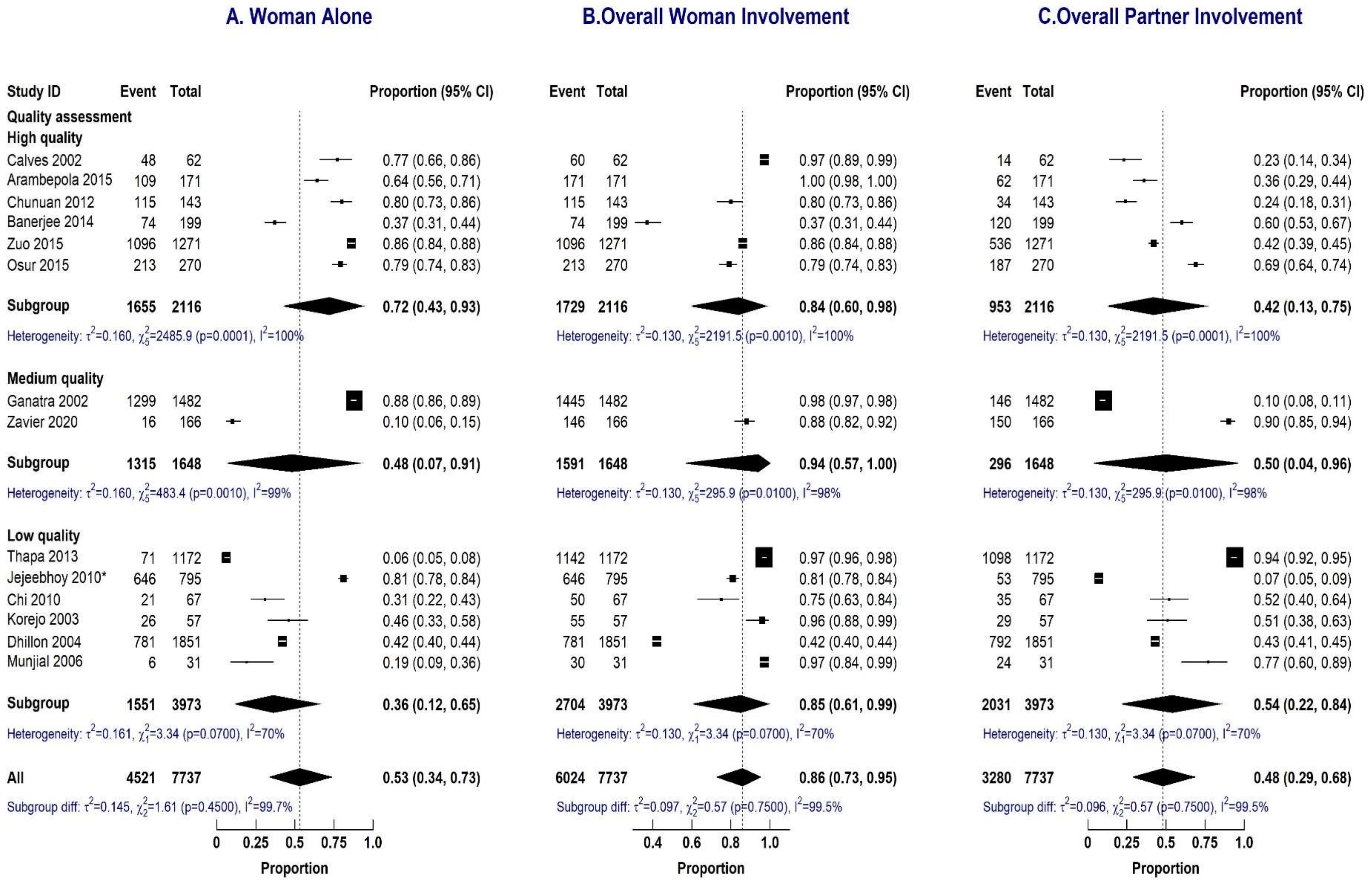

Supplement: Supplementary file 1 [file mmc1.pdf]
